# Phosphorylation and Chromatin Tethering Prevent cGAS activation During Mitosis

**DOI:** 10.1126/science.abc5386

**Published:** 2021-02-04

**Authors:** Tuo Li, Tuozhi Huang, Mingjian Du, Xiang Chen, Fenghe Du, Junyao Ren, Zhijian J. Chen

**Affiliations:** 1 Department of Molecular Biology, University of Texas Southwestern Medical Center, Dallas, Texas 75390-9148; 2 Center for Inflammation Research, University of Texas Southwestern Medical Center, Dallas, Texas 75390-9148; 3 Howard Hughes Medical Institute, University of Texas Southwestern Medical Center, Dallas, Texas 75390-9148

## Abstract

The cyclic GMP-AMP synthase (cGAS) detects microbial and self-DNA in the cytosol to activate immune and inflammatory programs. cGAS also associates with chromatin especially after nuclear envelope breakdown when cells enter mitosis. How cGAS is regulated during cell cycle transition is not clear. Here we found direct biochemical evidence that cGAS activity was selectively suppressed during mitosis, and uncovered two parallel mechanisms underlying this suppression. Firstly, cGAS was hyperphosphorylated at the N-terminus by mitotic kinases, including Aurora kinase B. The N-terminus of cGAS was critical for sensing nuclear chromatin, but not mitochondrial DNA. Chromatin sensing was blocked by hyperphosphorylation. Secondly, oligomerization of chromatin-bound cGAS, which is required for its activation,was prevented. Together, these mechanisms ensure that cGAS is inactive when associated with chromatin during mitosis, which may help to prevent autoimmune reaction.

## One Sentence Summary

cGAS is inhibited by phosphorylation and chromatin tethering during mitosis.

Cyclic GMP-AMP synthase (cGAS) detects DNA in the cytosol as a danger signal of invasion by microbial pathogens (1). Upon direct binding to double-stranded DNA, cGAS synthesizes the second messenger cyclic GMP-AMP (cGAMP) (2) in a process that involves a conformational change at the cGAS active site (3–6) and liquid-liquid phase separation (7). cGAMP binds and activates the endoplasmic reticulum-localized adaptor protein stimulator of interferon genes (STING) (2, 3, 8–12). cGAMP-bound STING activates the TANK-binding kinase 1 (TBK1) and interferon regulatory factor 3 (IRF3) through a phosphorylation-dependent mechanism (13–15). STING also activates nuclear factor-κB (NF-κB) that functions together with IRF3 to induce the production of Type I interferons and other inflammatory cytokines to control and eliminate infections (16, 17). Because cGAS is activated by DNA in a sequence-independent manner, excessive accumulation of self-DNA in the cytoplasm can activate cGAS to promote auto-inflammatory diseases and cellular senescence (18).

Accumulating data show that cGAS is tightly associated with chromatin during mitosis, a phase of the cell cycle when the nuclear envelope breaks down (19, 20). Following mitosis, a significant portion of cGAS remains chromatin-associated (19). How chromatin-associated cGAS is regulated remains poorly understood. Recent biochemical studies show that nucleosomes inhibit cGAS activity via tethering cGAS to the histone’s acidic patch (20–26). cGAS mutants defective in nucleosome-binding are de-repressed in cells, indicating that nucleosome-tethering potently blocks cGAS from sensing self-DNA (21). However, in biochemical assays nucleosomes only partially inhibit cGAS activity in the presence of linker DNA (20, 24, 26), which is likely abundant in cells, suggesting that additional mechanism of cGAS inhibition may exist. When mitosis breaks down the nuclear-cytoplasmic barrier, it is also unclear whether nuclear tethering is sufficient to overcome the impact of cytoplasmic cGAS.

## cGAS activity is selectively suppressed during mitosis

**Fig. 1.**
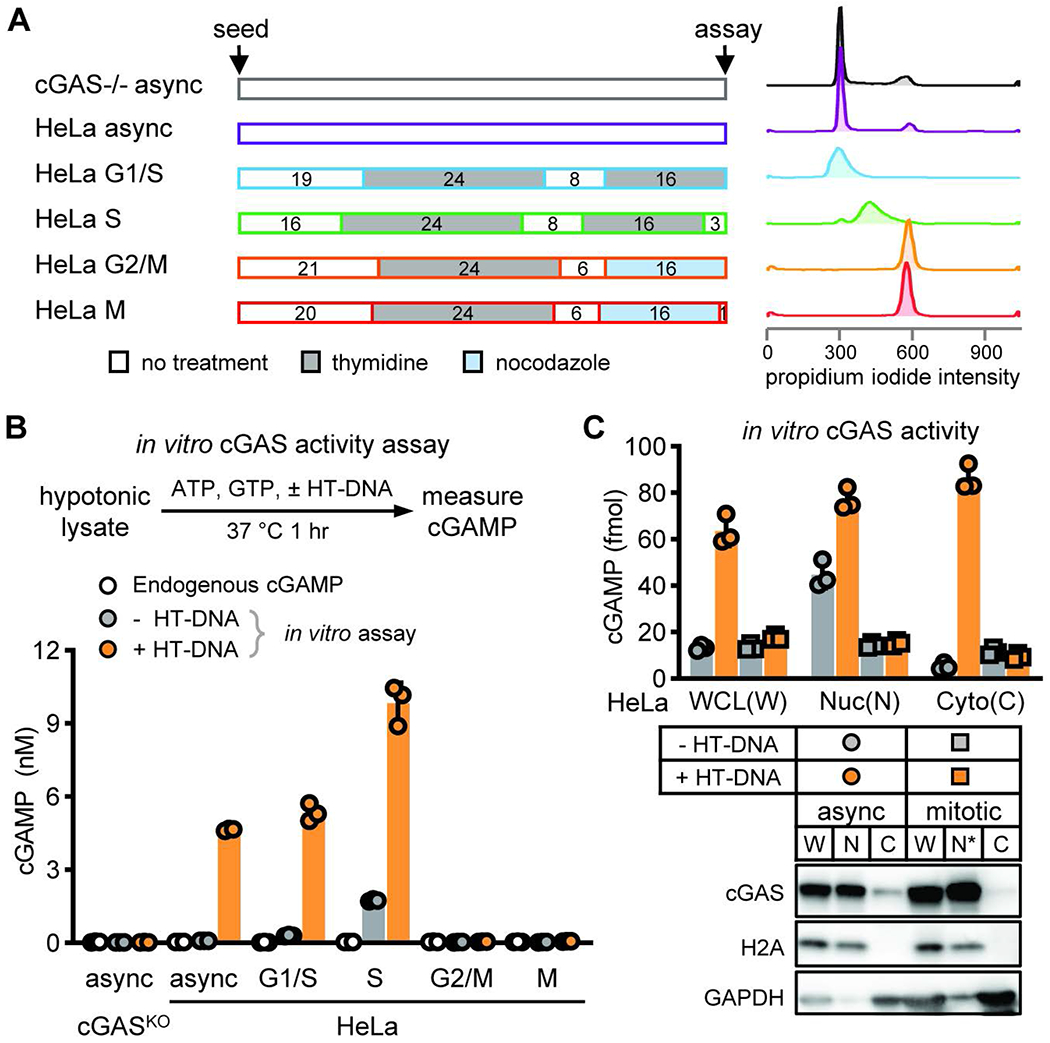
cGAS activity potential is selectively suppressed during mitosis **(A)** To obtain HeLa cells synchronized at different cell cycle stages, cells were treated with double thymidine blocks for G1/S and S phases, treated with thymidine followed by nocodazole for G2/M and M phases, or center untreated for asynchronized cells (denoted as async hereinafter). Schematic of the synchronization method (center). Numbers in boxes indicate duration of treatment in hours and color shades are types of treatment. Histograms of propidium iodide staining intensity indicate nuclear DNA contents in resulting cell populations (right). **(B)** cGAS activity at different cell cycle stages was measured as the amount of cGAMP produced from total hypotonic lysate incubated without (grey circles) or with (orange circles) herring testes DNA (HT-DNA). Endogenous cGAMP levels at different cell cycle stages was also measured from cell lysates before HT-DNA incubation (white circles). **(C)** Asynchronized or mitotic HeLa cells were lysed in hypotonic buffer and fractionated into indicated fractions: WCL (W), whole cell lysate; Nuc (N), nuclear; N*, mitotic chromatin; Cyto (C), cytoplasmic. cGAS activity was measured as the amount of cGAMP produced from these fractions incubated with or without HT-DNA. (B-C) Bars and errors are mean ± standard deviations (SD) of triplicate experiments (N=3).

To study the behavior of cGAS during the cell cycle, we synchronized HeLa cells at different phases of the cell cycle with thymidine and nocodazole and confirmed the cell cycle stages using propidium iodide staining followed by flow cytometry (Fig. 1A). Endogenous cGAMP levels were undetectable in all phases examined (Fig. 1B), suggesting that cGAS was not activated even when associated with chromatin (fig. S1A). We then tested whether cGAS at different cell cycle stages could be activated by exogenous DNA. HeLa cells synchronized at different cell cycle stages were lysed in a hypotonic buffer and the cell lysates were incubated with herring testes DNA (HT-DNA), ATP, and GTP at 37°C. cGAMP levels were measured by delivering the reaction products into a THP-1 Lucia cell line that secretes a luciferase whose expression is controlled by an interferon-inducible promoter. The cGAS in the lysates from asynchronized, G1/S phase, or S phase cells produced high levels of cGAMP in the presence of DNA. The cGAS in the G1/S or S phase cell lysates also produced low levels of cGAMP in the absence of DNA, possibly because the lysates contained DNA from thymidine-induced replication stress and micronuclei formation (27). Notably, the cGAS in the G2/M or M phase cell lysates could not be stimulated by DNA (Fig. 1B). Furthermore, we analyzed the cGAS activity potential in nuclear and cytoplasmic fractions of asynchronized and mitotic HeLa lysates, and measured cGAMP by liquid chromatography coupled mass spectrometry (LC-MS) (Fig. 1C). Regardless of the cell cycle, most cGAS was found to be nuclear, presumably in association with chromatin. While nuclear cGAS from asynchronized cells could be activated by exogenous DNA, the cGAS from mitotic cells could not. Thus, cGAS is inactive when associated with mitotic chromatin and chromatin-bound cGAS in mitotic cells cannot be activated by exogenous DNA.

## cGAS is hyperphosphorylated during mitosis

**Fig. 2.**
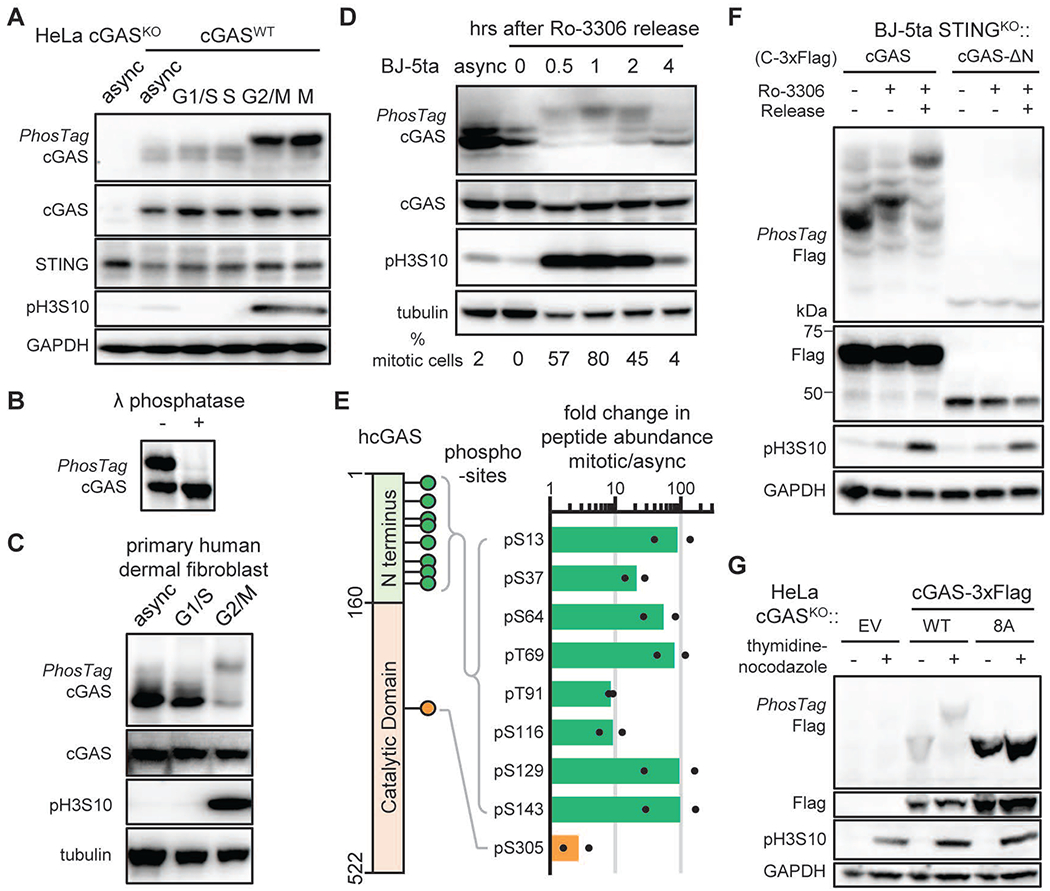
cGAS is hyperphosphorylated in the disordered N-terminus during mitosis **(A)** PhosTag electrophoresis indicates cGAS phosphorylation levels at different cell cycle stages. Immunoblotting of cGAS, STING, GAPDH, and Histone 3 S10 phosphorylation (pH3S10; a mitotic marker) is shown. **(B)** PhosTag electrophoresis and immunoblotting shows dephosphorylation of cGAS after lambda phosphatase treatment in hypotonic lysate of mitotic HeLa cells. **(C)** cGAS phosphorylation levels in synchronized primary human dermal fibroblasts. **(D)** Human BJ-5ta cells were synchronized with the reversible CDK1 inhibitor Ro3306 and released into cell cycle after removal of the inhibitor. PhosTag electrophoresis and immunoblotting show the phosphorylation status of cGAS and pH3S10, respectively. Numbers are percentage of mitotic cells measured by bright-field microscopy. **(E)** A schematic of the human cGAS sequence highlighting the phosphorylation sites observed by mass spectrometry (left). Fold changes in the abundance of phosphorylated peptides of cGAS in mitotic relative to asynchronized HeLa cells (right). Dots indicate data from two independent experiments and bars their mean values. **(F)** BJ-5ta STING^KO^ cells stably expressing cGAS or cGAS-ΔN were synchronized with 10 μM Ro3306 to the G2/M phase and released to mitosis. PhosTag electrophoresis and immunoblotting show the phosphorylation status of cGAS and pH3S10, respectively. **(G)** cGAS-deficient HeLa cells were reconstituted with wild type cGAS or the cGAS^8A^ mutant with all eight N-terminal phosphorylation sites replaced with alanine. These cells were arrested with thymidine and nocodazole before PhosTag electrophoresis. EV: empty vector.

To assess whether cGAS is regulated by phosphorylation during the cell cycle, we employed PhosTag electrophoresis, which detects phosphorylated proteins based on their slower mobility through the gel (28). PhosTag electrophoresis enabled us to observe slower migrating forms of cGAS only in nocodazole-arrested cells (Fig. 2A), which was sensitive to lambda phosphatase treatment (Fig. 2B), indicating that cGAS was phosphorylated during mitosis. To determine if cGAS is also phosphorylated in primary human cells, we performed similar analysis in primary human dermal fibroblasts. Like in HeLa cells, cGAS was also hyperphosphorylated when the fibroblasts entered the G2/M phase (Fig. 2C). To investigate cGAS phosphorylation using a different synchronization method, we treated BJ-5ta cells with a reversible CDK1 inhibitor, Ro3306, which blocks the cell cycle at the G2/M phase border, then released the cells into mitosis by removing Ro3306. After cell cycle blockage release, cGAS phosphorylation occurred as soon as 30 minutes, peaked at 1 hour, and disappeared at 4 hours. This correlated strongly with the levels of phosphorylated histone H3S10 and the percentage of cells entering mitosis (Fig. 2D). Thus, cGAS is phosphorylated during the mitotic phase of the cell cycle.

To locate the sites of cGAS phosphorylation, Flag-tagged cGAS was immuno-isolated from a stable HeLa cell line synchronized with nocodazole, digested with trypsin, and analyzed by LC-MS/MS shot-gun peptide sequencing. High-quality higher-energy collisional dissociation (HCD) spectra identified seven phosphorylated serine and two phosphorylated threonine residues in cGAS. Ser13, Ser37, Ser64, Thr69, Thr91, Ser116, Ser129, and Ser143 are within the intrinsically disordered amino terminus (N-terminus), whereas Ser305 is within the catalytic domain (Fig. 2E, fig. S2A, and Table S1). Parallel reaction monitoring (PRM) showed that phosphorylation of the N-terminal serine and threonine residues increased dramatically in mitotic cells compared to asynchronized cells, whereas phosphorylation of Ser305, previously shown to inhibit cGAS activity (29), did not change significantly in its abundance (Fig. 2E, fig. S3, Table S2). Removing the N-terminus of cGAS (1-160 amino acids) abolished the appearance of phosphorylated cGAS in the mitotic cell lysates when analyzed by PhosTag electrophoresis (Fig. 2F). Replacement of all eight phosphorylation sites with alanine (cGAS^8A^) also abolished the slower migrating form of cGAS shown by PhosTag electrophoresis (Fig. 2G), confirming that these sites largely account for the observed modification. Thus, cGAS is hyperphosphorylated at multiple serine and threonine residues within the N-terminus when cells enter mitosis.

## Mitotic hyperphosphorylation inhibits cGAS activity

**Fig. 3.**
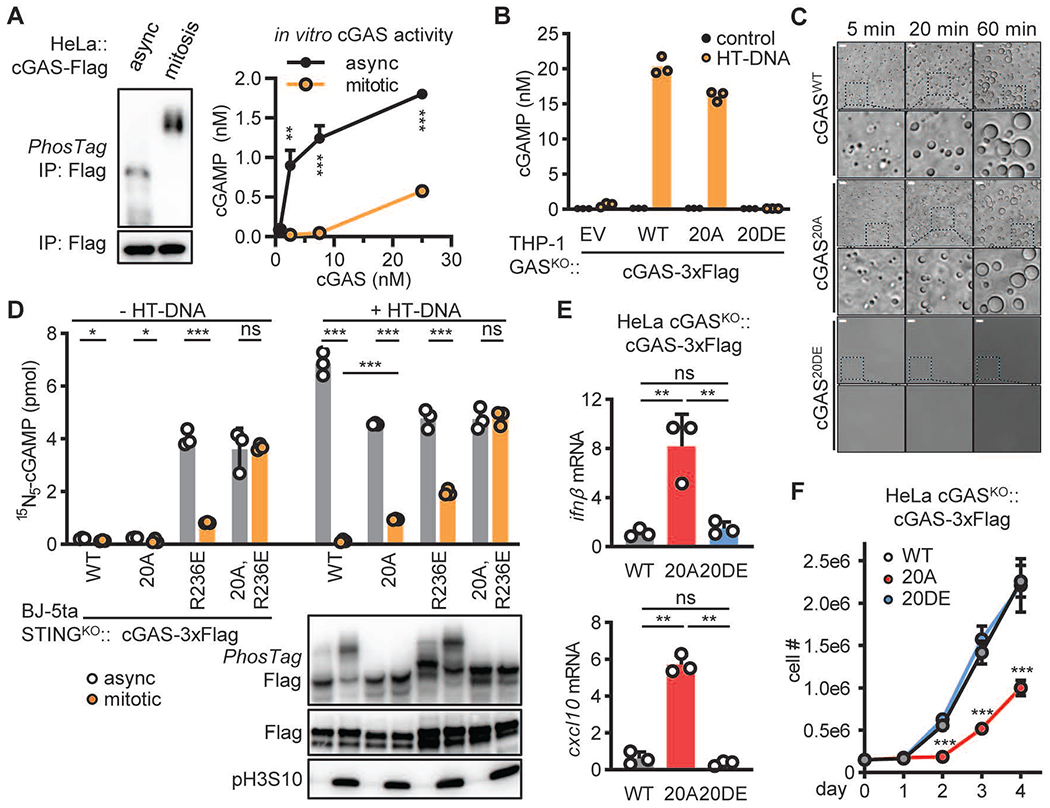
Mitotic hyperphosphorylation suppresses cGAS activity . (**A**) HeLa cells stably expressing cGAS-Flag were synchronized with thymidine and nocodazole or center untreated (async). cGAS-Flag proteins isolated from these cells were analyzed using PhosTag electrophoresis (left) or an in vitro enzymatic assay (right). (**B**) THP-1 cGAS^KO^ cells stably expressing cGAS^WT^, cGAS^20A^, or cGAS^20DE^ were transfected with HT-DNA. The level of cGAMP was measured at 4 hours after transfection. (**C**) 5 μM recombinant cGAS^WT^, cGAS^20A^, or cGAS^20DE^ was incubated with 5 μM 100 bp dsDNA in a physiological buffer at 37°C for the indicated time. Phase separation was visualized using bright field microscopy. Size bars = 10 μm. (**D**) STING-deficient BJ-5ta cells stably expressing Flag tagged cGAS^WT^, cGAS^20A^, cGAS^R236E^ or cGAS^R236E, 20A^ were synchronized with thymidine and nocodazole or center untreated (async). Hypotonic lysates of resulting cells were incubated with ATP, ^15^N_5_-GTP, and with or without HT-DNA at 37°C for 1 hr to measure the cGAS product ^15^N_5_-cGAMP with quantitative mass spectrometry (upper). PhosTag electrophoresis shows the level of cGAS phosphorylation and immunoblotting shows the level of pH3S10 (lower). (**E**) The levels of *ifnβ* and *cxcl10* RNA in HeLa cGAS^KO^ cells stably expressing C-terminally 3xFlag tagged cGAS, cGAS^20A^, or cGAS^20DE^ were quantified by RT-qPCR. (**F**) The growth curves of the cells shown in (E). (A, D-F) Data represent mean ± SD (N=3). Unpaired t-test: ns, not significant; *, p≤0.05; **, p≤0.01; *** p≤0.001.

The N-terminus of cGAS is disordered and highly enriched in positively charged lysine and arginine residues (fig. S2B), which mediate multivalent ionic interactions with DNA and promote the phase separation of cGAS and DNA into liquid droplets (7, 30). The identified mitotic phosphorylation sites of cGAS are evenly distributed across the entire N-terminus, approximately 13 amino acids apart. We hypothesized that phosphorylated serine or threonine residues might interfere through their evenly distributed negative charges with the ionic interactions between cGAS and DNA, thereby inhibiting cGAS activity. Indeed, cGAS isolated from mitotic HeLa cells was hyperphosphorylated and had markedly reduced activity compared to cGAS from asynchronized cells (Fig. 3A). Next, we replaced six serine and two threonine residues with aspartate (S>D) and glutamate (T>E) to mimic phosphorylation. However, the mutant protein cGAS^8DE^ was activated normally by DNA (fig. S4A). Because of the differences in side-chain acidity (pKa: 3.9 for D, 4.2 for E, and 1.2 for pSer or pThr), D/E substitutions have weaker electrostatic effect than phosphorylation. Increasing the number of S>D and T>E replacements progressively decreased the cGAS activity, and replacement of all N-terminal Ser and Thr residues with the acidic residues (cGAS^20DE^) largely abolished cGAS activity (Fig. 3B and fig. S4, A to D). In contrast, a cGAS mutant with alanine substitutions of all N-terminal Ser and Thr (cGAS^20A^) had similar activity to that of wild type (WT) cGAS (fig. S4, B to D). Moreover, both cGAS^20A^ and cGAS^WT^ proteins phase separated with DNA to form liquid droplets and catalyze cGAMP synthesis, whereas cGAS^20DE^ was unable to do so (Fig. 3C and fig. S4D). Together, our findings that phosphorylated cGAS from mitotic cells was much less active than the cGAS from asynchronized cells and that the cGAS^20DE^ mutant was much less active than the cGAS^20A^ mutant strongly suggest that hyperphosphorylation of the cGAS N-terminus inhibits its activity.

## Phosphorylation and chromatin tethering block cGAS activation during mitosis

We next tested the effect of hyperphosphorylation on cGAS activity during mitosis. HeLa cGAS knockout (cGAS^KO^) cells stably expressing cGAS^WT^ or cGAS^20A^ were synchronized by thymidine followed by nocodazole, and total cGAS activity was analyzed as above. The activity of both wild type cGAS and cGAS^20A^ was suppressed when cells were arrested at mitosis with nocodazole, although cGAS^20A^ produced significantly more cGAMP than cGAS^WT^ (Fig. 3D and fig. S5A). These results suggest that additional mechanisms exist besides phosphorylation to inhibit cGAS during mitosis. Nonetheless, we detected significant increases in the transcription of the cytokines *Ifnβ* and *Cxcl10* in HeLa cGAS^KO^ cells stably expressing cGAS^20A^ but not cGAS^WT^ or cGAS^20DE^ (Fig. 3E). In addition, HeLa cGAS^KO^ cells stably expressing cGAS^20A^ grew more slowly than those expressing cGAS^WT^ or cGAS^20DE^ (Fig. 3F). Thus, mitotic hyperphosphorylation plays an important role in suppressing cGAS activity.

Recent studies have shown that cGAS is strongly associated with chromatin in rapidly dividing cells *(19),* and that nucleosomes bind to cGAS through the acidic patch of H2A-H2B *(20, 22–26).* Mutations of Arginine 236 (R236) or R255 of human cGAS have been shown to weaken its tethering to chromatin and de-repress the enzyme (21). To test if chromatin tethering restricts cGAS activation during mitosis, we mutated R236 and R255 to glutamic acid (E) in cGAS^WT^ and cGAS^20A^. To avoid STING activation, we stably expressed these cGAS mutant proteins in BJ-5ta STING^KO^ cells. We prepared crude hypotonic lysates of cells grown asynchronously or synchronized at the mitotic phase and measured cGAS activity in the lysates in the presence or absence of HT-DNA. Because of the high level of endogenous cGAMP in these cells produced from the constitutively active cGAS mutants (fig. S5B), we incubated the lysates with ATP and ^15^N_5_-GTP to measure newly synthesized ^15^N_5_-cGAMP, which can be distinguished from natural cGAMP by mass spectrometry. Both cGAS^R236E^ and cGAS^R255E^ in asynchronous cell lysates were active even in the absence of exogenous DNA (Fig. 3D and fig. S5C), whereas purified cGAS^R236E^ and cGAS^R255E^ proteins were inactive in the absence of DNA (fig. S5D), suggesting that cell lysates contain chromosomal DNA that activates these cGAS mutants. Notably, cGAS^R236E^ and cGAS^R255E^ were still inhibited in mitotic cell lysates, and additional mutations in the N-terminal phosphorylation sites (20A) restored cGAS activation in these mitotic lysates (Fig. 3D and fig. S5C). Thus, both phosphorylation and chromatin tethering act in parallel to inhibit cGAS during mitosis.

## Aurora kinase B phosphorylates cGAS during mitosis

**Fig. 4.**
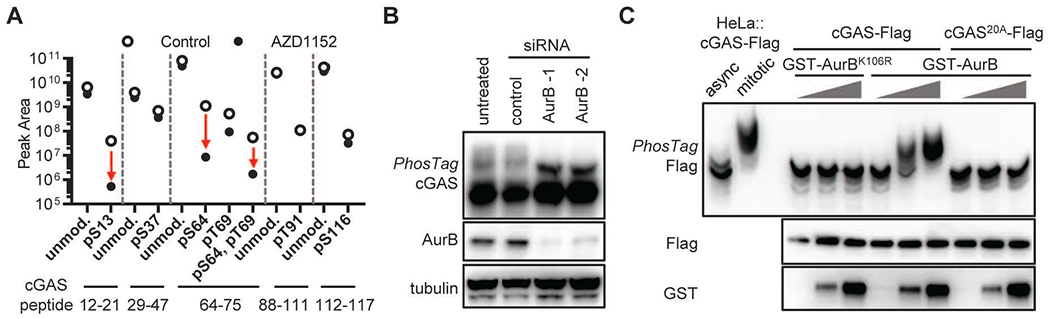
Aurora kinase B phosphorylates cGAS during mitosis (**A**) BJ-5ta cells stably expressing cGAS-Flag were synchronized with Ro-3306 and released into mitosis in the absence or presence of aurora kinase B inhibitor AZD-1152 (1 μM). cGAS-Flag was isolated from the cells to quantify the level of phosphorylation by mass spectrometry. The abundance (peak area) of phosphorylation at indicated sites was quantified by PRM to identify those sensitive to AZD-1152: arrows indicate large drops from untreated (∘) to AZD-1152-treated (•) samples. (**B**) BJ-5ta cells were transfected with siRNA targeting Aurora kinase B (AurB) or control siRNA, or untreated for 48 hrs before synchronization with thymidine and nocodazole treatment. The level of cGAS hyperphosphorylation in mitotic cells was analyzed by PhosTag electrophoresis and immunoblotting. (**C**) in vitro kinase assay with recombinant cGAS-Flag and GST-AurB. cGAS phosphorylation was analyzed by PhosTag electrophoresis. The phosphorylation status of endogenous cGAS in asynchronized and mitotic HeLa cells is shown on the same blot. K106R is a mutation that inactivates Aurora kinase B.

The phosphorylation sites of the cGAS N-terminus are surrounded by diverse sequences, which are likely recognized by more than a single kinase. In our search for the kinases that phosphorylate cGAS during mitosis, we noticed that the kinetics of cGAS modification strongly correlated with histone H3S10 phosphorylation, a well-known target of Aurora kinase B (AurB) (31). To test the role of aurora kinases in cGAS hyperphosphorylation, we treated BJ-5ta cells with Aurora kinase inhibitors (AMG-900, MLN-8237, or AZD-1152) after cells exited the G2/M arrest induced by Ro-3306. All three inhibitors abrogated H3S10 phosphorylation while still allowing cells to enter mitosis (fig. S6, A to B). Both cGAS hyperphosphorylation and H3S10 phosphorylation levels were reduced by aurora kinase inhibitors in a dose-dependent manner. In contrast, Polo-like kinase inhibitors BI 2536 or Volasertib did not affect cGAS phosphorylation (fig. S6A). Mass spectrometry showed that cGAS phosphorylation at Ser13 and Ser64 was significantly reduced in AZD-1152 treated mitotic cells, while other phosphorylation sites were not affected (Fig. 4A). These two sites also comply with the AurB substrate consensus: [R/K]XpS (32). The lowest effective concentration at which these aurora kinase inhibitors reduced cGAS hyperphosphorylation (AZD-1152_0.2μM_<AMG-900_0.5μM_<MLN-8237_3μM_) correlated with their potency against AurB (IC_50_: AZD-1152_1nM_<AMG-900_4nM_<MLN-8237_1μM_), but not with that against Aurora kinase A (IC_50_: MLN-8237_1μM_<AZD-1152_1.4μM_ <AMG-900_5μM_) (33–35). This suggests that it is AurB that specifically phosphorylates cGAS during mitosis. Like chemical inhibitors, siRNA-mediated knockdown of AurB also decreased cGAS hyperphosphorylation (Fig. 4B). Moreover, recombinant GST-AurB, but not the inactive mutant GST-AurB^K106R^ or cGAS^20A^, directly phosphorylated cGAS in vitro, (Fig. 4C). Thus, AurB phosphorylates cGAS at the N-terminus during mitosis. Upon exit from mitosis, dephosphorylation of cGAS may be mediated by protein phosphatase PP1 or PP2A, because inhibition of these enzymes with the inhibitor calyculin A led to robust cGAS phosphorylation in various cell types (fig. S6C).

## cGAS tethered to mitotic chromosomes cannot oligomerize

**Fig. 5.**
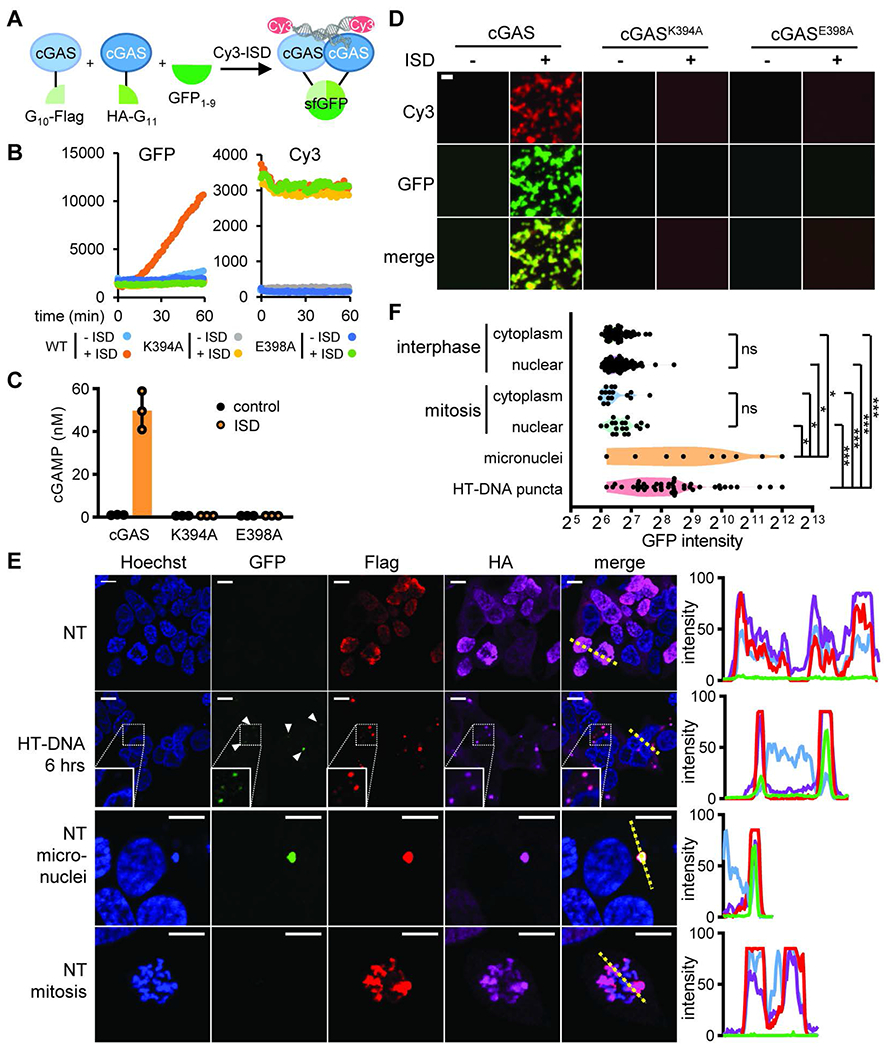
Chromatin-bound cGAS is prevented from oligomerization (**A**) Schematic of the tripartite split GFP reporter for cGAS activation. cGAS is fused at the N-terminus with the G10 fragment and the Flag tag or at the C-terminus with the HA tag and the G11 fragment. In the presence of the detector fragment GFP1-9 and double stranded DNA (Cy3 labelled Interferon stimulatory DNA, ISD), cGAS oligomerization allows GFP fragment complementation to generate green fluorescence. (B-D) Purified components of the split GFP system were mixed in vitro. K394A and E398A are two mutations that interfere with cGAS dimerization. (**B**) Fluorescence intensities in the GFP and Cy3 channels measured by the plate reader over time; (**C**) cGAMP levels measured at the end of incubation; (**D**) Fluorescent microscopy showing cGAS-DNA phase separation. (**E**) Representative fluorescence microscopy images showing the level and distribution of GFP in HEK293T cells stably expressing G10-Flag-cGAS, cGAS-HA-G11, and GFP1-9 (left). Immunofluorescence via Flag and HA indicates the expression of cGAS fusion proteins. Intensity profiles in each channel along highlighted yellow dotted lines in the merged images are shown (right). (D and E) Size bars = 10 μm. (**F**) Scattered plot of GFP intensity measured from indicated subcellular areas of the split GFP HEK193T cells as shown in (E): cytoplasm or nuclei of cells during interphase or mitosis, micronuclei in untreated cells, and DNA puncta observed in HT-DNA transfected cells. Unpaired t-test between indicated sample groups: ns, not significant; *, p≤0.05; *** p≤0.001.

To better understand the status of cGAS activation during the cell cycle, we implemented a split green fluorescence protein (GFP) reporter system. The super fold GFP (sfGFP) was split into three complementary fragments: β-strand 10 (GFP10), β-strand 11 (GFP11), and β-strand 1-9 (GFP1-9) (36). The two smaller fragments were fused to the N- or C- terminus of cGAS, respectively, to create two cGAS constructs that may complement each other if cGAS dimerizes or oligomerizes (Fig. 5A). When the three purified components were mixed in vitro, the addition of DNA stimulated GFP formation, cGAMP production, and cGAS-DNA phase separation (Fig. 5, B to D).

Importantly, cGAS activity and phase separation were abolished by mutations that disrupt the dimer interface, K394A or E398A (6), providing strong evidence that cGAS dimer formation is required for cGAS phase separation and activation. Thus, the tripartite split GFP reporter is a good indicator of cGAS oligomerization, phase separation, and activation. When stably overexpressed in HEK293T cells, the split GFP reporter did not exhibit any GFP signal in unstimulated cells (Fig. 5, E to F). After transfection with HT-DNA, GFP puncta appeared in the cytoplasm, indicating cGAS oligomerization and activation. In cells arrested in mitosis during which cGAS was tethered to chromatin, GFP fluorescence in chromatin regions was undetectable, suggesting that chromatin-bound cGAS was prevented from oligomerization. Previous studies have shown that in cells containing micronuclei, cGAS can enter the micronuclei and be activated by chromatin fragments (19, 37–41). Consistent with these reports, some cGAS-containing micronuclei in the cytoplasm showed high GFP fluorescence (Fig. 5, E to F). Thus, cGAS in the primary nuclei, but not micronuclei, cannot form oligomers while being chromatin-tethered, providing an explanation for the suppression of cGAS activity during mitosis.

## The N-terminus of cGAS is required for sensing chromatin DNA but not mitochondrial DNA

**Fig. 6.**
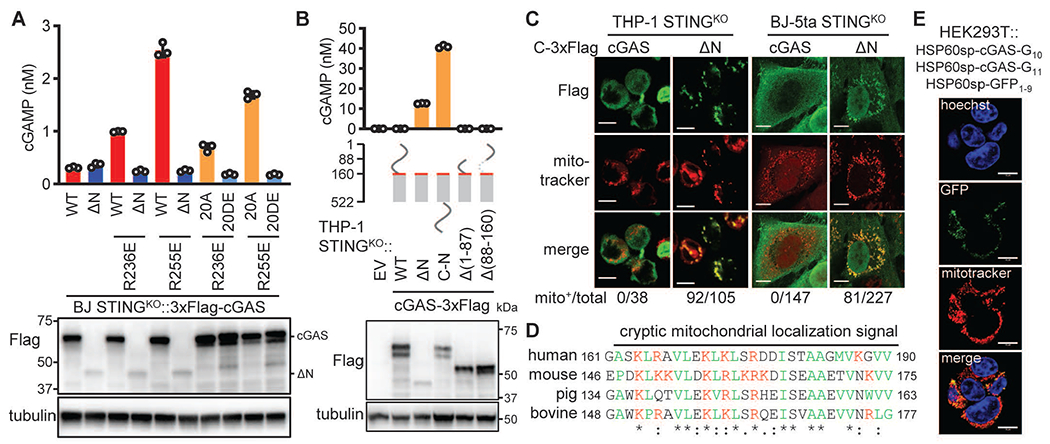
The cGAS N-terminus is required for sensing chromatin DNA and for preventing cGAS activation in the mitochondria (**A**) BJ STING^KO^ cells stably expressing WT cGAS or the indicated mutants were analyzed for endogenous cGAMP levels (upper). Cell lysates were analyzed by immunoblotting for the indicated proteins (lower). (**B**) THP-1 STING^KO^ cells stably expressing empty vector (EV) and indicated cGAS proteins: endogenous cGAMP levels and schematic of constructs (upper). Red lines, cryptic mitochondrial localization sequence (MLS). Immunoblotting showing expression of cGAS proteins depicted in the middle diagrams (lower). (**C**) Confocal microscopy images showing distinct subcellular distribution of cGAS and C-Flag cGAS-ΔN in THP-1 STING^KO^ or BJ-5ta STING^KO^ cells in relation to mitochondria (mitotracker). Values shown on the bottom are the numbers of cells displaying mitochondrial cGAS relative to total numbers of cells examined. **(D)** Alignment of the MLS of cGAS from different species. Red, positively charged residues; green, hydrophobic residues. (**E**) The split GFP-cGAS proteins were fused at the N-termini with the signal peptide from Hsp60, which targets the proteins to the mitochondrial matrix. Confocal microscopy shows GFP distribution in HEK293T cells stably expressing these proteins. Size bars in (C and E) = 10 μm.

To investigate the role of cGAS N-terminus in sensing chromatin DNA, we deleted this region from wild type cGAS as well as the cGAS^R236E^ and cGAS^R255E^ mutants. When stably expressed with an N-terminal 3×Flag tag (N-Flag) in BJ STING^KO^ cells, cGAS-ΔN^R236E^ and cGAS-ΔN^R255E^ did not cause constitutive cGAMP production, unlike the full-length cGAS^R236E^ and cGAS^R255E^ (Fig. 6A). As a control, cGAS-ΔN and cGAS-ΔN^R255E^ could still respond to transfected DNA (fig. S5E). The data suggest that the N-terminus of cGAS is critical for these mutants to sense chromatin DNA. In addition, the 20DE mutation inactivated cGAS^R236E^ and cGAS^R255E^, whereas the 20A mutation restored the activity (Fig. 6A), indicating that hyperphosphorylation in the N-terminus prevents cGAS activation by chromatin DNA. The lower levels of cGAS-ΔN^R236E^ or cGAS-ΔN^R255E^ is unlikely the reason for loss of activity, because cGAS-ΔN tagged with a C-terminal 3×Flag tag (C-Flag) was constitutively active when stably expressed in the THP-1 STING^KO^ cells or the BJ-5ta STING^KO^ cells; the expression level of C-Flag cGAS-ΔN was comparable to that of N-Flag cGAS-ΔN and both levels were lower than their full-length counterpart (Fig. 6B and fig. S7, A to B). Overexpression of C-Flag cGAS-ΔN was not tolerated in THP-1 cGAS^KO^ or BJ-5ta cGAS^KO^ cells, both of which express STING, whose activation led to cell death. Consistent with this, the surviving cells were found to lack cGAS-ΔN (fig. S7, C to D). Thus, the disordered N-terminus is critical for cGAS chromatin DNA sensing and this sensing is blocked by mitotic hyperphosphorylation

We further aimed to understand the mechanism underlying the constitutive activation of cGAS-ΔN, which has also been observed by others (42). It has been proposed that the N-terminus tethers cGAS to the plasma membrane, thereby inhibiting its activity (42). We noticed that N-Flag cGAS-ΔN was not active (Fig. 6A and fig. S7A), which suggests that deletion of the N-terminal sequence is not sufficient for activation. Notably, appending the N-terminal region to the C-terminus of cGAS-ΔN led to strong activation, whereas removing residues 1-86 or 87-160 from cGAS did not. Upon closer examination, we found that cGAS-ΔN could localize to the mitochondria (Fig. 6C).

cGAS-ΔD was significantly more protected from trypsin digestion in mitochondria-containing fractions than full-length cGAS (Fig. S8A), supporting the idea that cGAS-ΔN is in the mitochondrial matrix. Indeed, cGAS-ΔN has in its N-terminus a cryptic mitochondrial targeting sequence (MTS), which is enriched in positively charged residues followed by hydrophobic residues (Fig. 6D). Furthermore, fusion of a 30 amino acid peptide (161-190 in full length cGAS), but not a preceding peptide (136-160 in cGAS), targeted GFP to the mitochondria (fig. S8B). A shorter peptide (161-175 from full length cGAS) could also target GFP to the mitochondria, albeit less efficiently. Consistent with the difference in activation, only C-Flag, not N-Flag tagged cGAS-ΔN showed prominent mitochondrial localization (fig. S8C), likely because extra N-terminal sequences block the cryptic signal peptide function. Mitochondrial localization was also observed for C-terminally HA-tagged cGAS-ΔN (fig. S8D).

To test if cGAS-ΔN is activated by mitochondrial DNA (mtDNA) in the matrix, we expressed the Y147A uracil-N-glycosylase mutant (UNG) to degrade mtDNA in THP-1 STING^KO^::cGAS-ΔN cells (43). The Y147A mutation enables the uracil-cutting enzyme to also cut thymine, thereby enabling depletion of mtDNA. The mtDNA-depleting treatment also significantly reduced cGAMP levels, supporting the idea that activation of cGAS-ΔN is mtDNA-dependent (fig. S8E). To further examine the potential of the mitochondrial genome in activating cGAS, we targeted the tripartite split-GFP-cGAS reporter system to the mitochondrial matrix by fusing the signal peptide of HSP60 to all three components. High GFP fluorescence was observed as a result of mitochondrial localization of cGAS (Fig. 6E), confirming that mtDNA is a potent cGAS ligand, unlike nuclear chromatin. The mitochondrial transcription factor A (TFAM) has been shown to promote DNA bending and cGAS activation (44). Consistent with these results, we found that TFAM promoted cGAS-DNA phase separation (fig. S8F). Thus, the cGAS N-terminus blocks a cryptic MTS from delivering cGAS to the mitochondria, which would otherwise lead to cGAS activation by mtDNA.

Finally, we tested whether cGAS could be activated by endogenous mtDNA that is released to the cytosol after cells are treated with the Bcl-2 inhibitor (ABT-737) and a caspase inhibitor (Q-VD-PQH), which has been reported to cause cGAS activation (45). The N-terminal Flag-tagged cGAS proteins, including cGAS-ΔN^R255E^, were all activated when cells were treated with the compounds that caused the release of mtDNA to the cytosol (fig. S8G). Thus, the N-terminus of cGAS is required to sense chromatin but not mitochondrial genomic DNA and the N-terminal phosphorylation of cGAS blocks its activation by chromatin DNA.

## Discussion

Cells must stringently regulate cGAS activity in order to mount a robust immune defense against infections while avoiding autoimmunity. The tight association of cGAS with chromatin, especially during mitosis, raises an important question of how cGAS activity is regulated during the cell cycle. Here we present direct evidence that cGAS activity is suppressed during mitosis through two mechanisms, hyperphosphorylation at the N-terminus and inhibition of oligomerization due to chromatin tethering. Both mechanisms prevent cGAS phase separation into liquid droplets where cGAS can efficiently synthesize cGAMP. Our data also reveal distinct roles of the cGAS N-terminus in sensing nuclear chromatin and mitochondrial DNA; cGAS depends on an unphosphorylated N-terminus to form multivalent interactions with the nuclear chromatin DNA, whereas mitochondrial genomes can promote cGAS oligomerization with the help of DNA-bending proteins such as TFAM (44). Hyperphosphorylation at the N-terminus is therefore an in-cis mechanism to inactivate cGAS, while nuclear tethering acts in trans. While chromatin-tethering can inhibit cGAS activity during interphase, mitosis presents a unique challenge because of an influx of cytoplasmic cGAS after nuclear envelope breakdown. This dual-layer mechanism of inhibition ensures that cGAS is not aberrantly activated by chromatin DNA throughout the cell cycle.

## Materials and Methods

**Table:** 

Reagents	Source	Identifier
**Chemicals**		
Thymidine	Sigma	T9250
Nocodazole	Sigma	M1404
Ro-3306	Selleckchem	S7747
Tozasertib (VX-680)	Selleckchem	S1048
BI 2536	Selleckchem	S1109
Volasertib (BI 6727)	Selleckchem	S2235
ABT-737	Selleckchem	S7311
Q-VD-OPH	Selleckchem	S1002
AMG-900	Cayman Chemical	19176
MLN-8237	Cayman Chemical	13602
AZD-1152-HQPA	Cayman Chemical	11602
Calyculin A	Cayman Chemical	19246
PhosTag	Wako Chemicals	304-93521
Lambda phosphatase	New England Biolabs	P0753S
Mitotracker Red	Thermo Scientific	M7513
Herring sperm DNA	Thermo Scientific	15634017
Lipofectamine 2000	Thermo Scientific	11668019
Hoechst 33342	Thermo Scientific	62249
**Antibodies**		
rabbit anti-cGAS mAb	Cell Signaling	Clone D1D3G
rabbit anti-STING mAb	Cell Signaling	Clone D2P2F
rabbit anti-pIRF3	Cell Signaling	Clone D601M
rabbit anti-GAPDH mAb	Cell Signaling	Clone D16H11
rabbit anti-Hsp60	Cell Signaling	Clone D6F1
rabbit anti-Tom20	Santa cruz	SC11415
rabbit anti-pH3S10 mAb	Bethyl	A301-844A-T
mouse anti-Flag mAb	Sigma	F3165
mouse anti-tubulin mAb	Sigma	Clone DM1A

## Cell culture

HeLa, BJ-5ta, HEK293T, and human primary dermal fibroblast cell lines (ATCC, PCS-201-010) are maintained in Dulbecco’s modified Eagle’s medium (Thermo Scientific) supplemented with 10% fetal bovine serum (Sigma) and 1% penicillin and streptomycin. THP-1(ATCC) and THP-1 Lucia ISG (Invivogen) cell lines are maintained in RPMI-1640 medium (Gibco, Thermo Scientific) supplemented with 10% fetal bovine serum, 0.05mM 2-mercaptoethanol (Gibco, Thermo Scientific), and 1% penicillin and streptomycin.

All cells were cultured in 37°C humidified incubators with 5% CO_2_.

## Cell cycle synchronization

To synchronize HeLa cells at the G1/S phase, cells were cultured with 2.5 mM thymidine for 24 hours (hr), washed and then cultured in fresh media for 8 hr before incubating again with thymidine for 16 hr. To synchronize cells at the S phase, cells arrested at the G1/S phase were released into thymidine-free medium for 3-4 hr. To synchronize cells at the G2/M phase, cells arrested by 24-hr thymidine treatment were cultured in fresh medium for 8 hr and then incubated with 100 nM nocodazole for 16 hr. To obtain M phase cells, cells arrested at the G2/M phase were cultured in nocodazole-free medium for 1 hr. Cells were also synchronized at the G2/M border with 10 μM CDK1 inhibitor Ro-3306 (Sigma) for 16 hr, washed and then incubated in fresh medium; these cells were collected at 0, 0.5, 1, 2, and 4 hours after fresh media incubation for analysis.

## in vitro cGAS activity assay

Cells were lysed in a hypotonic buffer (10 mM Tris-HCl, pH 7.4, 5 mM MgCl_2_) with 1 min vigorous mixing by plastic pellet pestle in 1.5 mL tubes. The protein concentration of lysates was measured with Commassie Plus protein assay reagent (Thermo Scientific), using bovine serum albumin (BSA) as the standard. 300 μg total protein was used for cGAS activity assays in a reaction mixture of 20 mM Tris-HCl, pH 7.4, 5 mM MgCl2, 0.2 mg/mL BSA, 10 ng/μL HT-DNA, 1 mM ATP, and 1 mM GTP. After 1 hr incubation at 37°C, samples were boiled for 10 min and centrifuged at 20,000 g for 5 min. The resulting supernatant was used to measure cGAMP levels using mass spectrometry or the activity assay that are described below. The activity of cGAS proteins purified from *E.coli* or HeLa cells was measured similarly. For testing the cGAS^R236E^ and cGAS^255E^ mutants expressed in BJ-5ta STING^KO^ cells, the same in vitro tests were performed, except that ^15^N_5_-GTP (Sigma, 707775) was used instead of regular GTP. The level of ^15^N_5_-cGAMP was measured by mass spectrometry as described below.

## cGAS-DNA liquid phase separation assay

To assess cGAS-DNA phase separation, 5 μM of recombinant cGAS protein was incubated with 5 μM of 100 bp dsDNA in a buffer containing 20 mM Tris-HCl, pH 7.4, 5 mM MgCl_2_ at 37°C for the indicated period of time. At the end of incubation, the formation of liquid droplets was observed under light microscopy. The sequence of the forward strand of the 100 bp dsDNA is ACATCTAGTACATGTCTAGTCAGTATCTAGTGATTATCTAGACATACATCTAGTACATGTCTAGTCAGTATCTAGTGATTATCTAGACATGGACTCATCC.

## Measurement of cGAMP levels using the THP-1 Lucia ISG reporter cells or mass spectrometry

To measure cGAMP levels using THP-1 Lucia ISG cells, 2.5×10^5^ reporter cells were seeded in a 96-well plate in 50 μL serum-free DMEM supplemented with 50 ng/mL recombinant Perfringolysin O (PFO). 5 μL supernatant of heated lysate supernatant (see above) was added to each well for 16-hr at 37°C. 20 μL of this culture was transferred into a 100 μL CZ buffer containing 1 μM coelenterazine (Gold Biotechnology), 50 mM Tris-HCl, pH 7.0, 50 mM NaCl, 20 mM EDTA, and 20% glycerol. Luminescence was measured in an opaque 96-well plate with a CLARIOstar microplate reader (BMG LABTECH). Pure cGAMP (0.3 nM to 100 nM) was used to obtain a standard curve.

To measure cGAMP by LC-MS, hydrophilic metabolites and 80 fmol spiked-in internal standard (^15^N_10_-cGAMP, in-house generated) were extracted from cell pellets, subsequently in 80% methanol and 2% acetic acid, and twice in 2% acetic acid. cGAMP was enriched from combined extracts on HyperSep Aminopropyl SPE Columns (Thermo Scientific). After washing twice in 2% acetic acid and once in 80% methanol, samples were eluted in 4% ammonium hydroxide in 80% methanol. Vacuum-dried eluents were dissolved in water and analyzed on a Dionex U3000 HPLC coupled with TSQ Quantiva Triple Quandruple mass spectrometer (Thermo Scientific). The Nano LC stationary phase is a LUNA NH_2_ resin (5 μm, Phenomenex) packed in 0.1 mm ID × 70 mm L silica capillaries with an emitter tip fabricated on a laser capillary puller (Sutton P-2000). Mobile phases are acetonitrile (A), and 20 mM ammonium bicarbonate and 20 mM ammonium hydroxide aqueous solution (B). Flow rate is 800 nL/min (0-4 min), 300 nL/min (4-19 min), and 600 nL/min (19-27 min), with a gradient of 20% B (0-3 min), 50% B (4 min), 80% B (14-18 min), and 20% B (19-27 min). Nano LC eluent was directly ionized with a Nanospray Flex Ion source (Thermo Scientific), with +2000 V spray voltage, 340°C for ion transfer tube temperature, and 0 units of sweep gas. Multiple reaction monitoring was performed in the positive mode at 50 ms dwell time, 115 V RF lens, 0.4 FWHM in Q1 and Q3 resolution, 1.5 mTorr CID gas, 10 V in source fragmentation, and 3 sec chrom filter. Transitions were 675-136, 675-152, 675-476, and 675-524 for cGAMP; 685-136, 685-157, 685-480, and 685-529 for the ^15^N_10_-cGAMP standard; and 680-136, 680-157, 680-476, and 680-524 for ^15^N_5_-cGAMP. Raw data was converted in ReAdW to the mzXML format, followed by noise reduction and processing in MATLAB. Endogenous cGAMP levels were caculated by multiplying the cGAMP-to-standard ratios by 80 fmol, the amount of standard spiked into each sample.

## PhosTag SDS–PAGE electrophoresis and lambda phosphatase treatment

Phosphorylation of cGAS was analyzed by PhosTag SDS-PAGE electrophoresis. Cell pellets were resuspended in Laemmli buffer and boiled for 10 min. Proteins were separated on 10% SDS-PAGE gels supplemented with 50 μM PhosTag AAL (Wako) and 50 μM MnCl2 in an XCELL SureLOCK Mini-Cell (Invitrogen) until the dye front escaped. Mn^2+^ was chelated from gels by 10-min washes in transfer buffer supplemented with 1 mM EDTA. After three times of water rinse, gels underwent semiwet protein transfer onto a PVDF membrane and standard immunoblotting was subsequently performed to detect cGAS. To dephosphorylate cGAS, the hypotonic lysate of mitotic HeLa cells was incubated with lambda phosphatase in the presence of 1 mM MnCl_2_ at 30°C for 30 min. Lysates were denatured in Laemmli buffer at 95°C for 10 min, and analysed by PhosTag electrophoresis and immunoblotting.

## Mapping the cGAS phosphorylation sites by mass spectrometry

Human cGAS with a C-terminal Flag tag was stably expressed in HeLa cGAS^KO^ cells. Cells were synchronized by Ro-3306 and released into mitosis for 1 hr. Mitotic cells were washed off the plate, collected by centrifugation, washed in PBS, and lysed in ice cold hypotonic buffer plus 0.5% NP-40. The lysate was centrifuged at 2,000 g for 5 min to obtain the P2 fraction, which was treated with 10 unit/mL of benzonase (Sigma) on ice for 10 min. After centrifugation at 2,000 g for 5 min, the pellet was extracted in a high salt buffer containing 20 mM Tris-HCl, pH 7.4, 500 mM NaCl on ice for 10 min. After centrifugation at 10,000 g for 10 min, the supernatant contained the majority of cGAS, which was then immuno-isolated on magnetic Dynabeads M-270 Epoxy conjugated with M2 anti-Flag antibody (Sigma). After incubation at 4°C for 1 hr with head-to-end rotation, the magnetic beads were isolated from the cell supernatant on a magnetic rack and washed for six times with high salt buffer plus 0.5% NP-40. cGAS-Flag was eluted off beads in 1× LDS buffer (Thermo Scientific) by heating at 70°C for 10 min.

Immuno-purified cGAS proteins were separated on 4–12% NuPAGE Novex Bis-Tris gels (Thermo Scientific) and visualized with SimplyBlue SafeStain (Thermo Scientific). Protein bands corresponding to cGAS were excised and destained in 50 mM ammonium bicarbonate (ABC) and 50% acetonitrile (ACN) for 10 min. Gel slices were dehydrated in 100% ACN and rehydrated in 50 mM ABC plus 5 mM DTT for 30 min. 50 mM lodoacetamide was added for 60 min. Gel pieces were dehydrated, rehydrated in ABC, and additionally dehydrated before overnight incubation with 12.5 ng/μL trypsin (Promega) in 50 mM ABC at 37°C. The resulting peptides were extracted in 1% formic acid (FA) at 25 °C for 4 hr and then in 0.5% FA/0.5% ACN for 2 hr. After acidification in 1% formic acid, peptide mixtures were further purified with C18 Zip-tip (Millipore) and analyzed by nano–liquid chromatography–mass spectrometry (nLC-MS).

Peptide mixtures were separated on an in-house packed C18 column in silica capillary emitters (resin: 100 Å, 3 μm, MICHROM Bioresources; column: 100 μm ID, 100 mm resin length). A Dionex Ultimate 3000 nanoLC system (Thermo Scientific) provided the LC gradient with 0.1% formic acid as mobile phase A and 0.1% formic acid in acetonitrile as mobile phase B. The following gradient was used: 2% B at 0–15 min, 30% B at 81 min, 35% B at 85 min, 40% B at 87 min, 60% B at 95 min, 80% B at 96μ107 min, and 2% B at 108–120 min. Flow rate was 600 nL/min at 0–13.5 min and 250 nL/min at 13.5–120 min.

Peptide eluents were sprayed online with a nano-electrospray ion source (Thermo Scientific) at a spray voltage of 1.5 kV and a capillary temperature of 250°C. High-resolution MS analysis was performed on a QExactive HF-X Quadrupole-Orbitrap Hybrid mass spectrometer (Thermo Scientific), operating in data-dependent mode with dynamic exclusion of 30 s. Full-scan MS was acquired at an m/z range of 300–1650, resolution of 70,000, and automatic gain control target of 3×10^6^ ions. The top 15 most intense ions were subsequently selected for higher-energy collisional dissociation (HCD) fragmentation at a resolution of 17,500, collision energy of 30 eV, and automatic gain control target of 1×10^5^.

Peptide-spectrum matches were performed by the SEQUEST algorithm in Proteome Discoverer (Thermo Scientific), using the human proteome database (Uniprot, UP000005640) plus common contaminants. Static modification: Cys carbamidomethylation; variable modifications: Ser or Thr phosphorylation, Met oxidation, and Gln or Asn deamination; precursor mass error: 10 ppm; fragment mass error: 0.05 Da; maximum mis-cleavage: 2; peptide false discovery rate: 1%. SEQUEST results were refined by the X!Tandem algorithm using Scaffold (version 4.6.1; Proteome Software), and cGAS phosphorylation sites were scored in Scaffold PTM.

## Quantification of cGAS phosphorylation by parallel reaction monitoring (PRM)

To quantify levels of cGAS phosphorylation during the cell cycle transition, we isolated Flag-tagged cGAS from HeLa cells that were synchronized by thymidine and nocodazole, or not synchronized. cGAS was separated by PhosTag electrophoresis and visualized by Commassie staining. The cGAS band from asynchronized cells and the upper band from synchronized cells were excised for trypsin digestion. Resulting peptides were separated by reverse phase chromatography as above and analyzed by parallel reaction monitoring (PRM) that repeatedly select prominent ions of phosphorylated peptides and their nonphosphorylated counterparts for HCD fragmentation on a QExactive HF-X mass spectrometer (Thermo Scientific). PRM parameters were resolution 60,000, AGC target 5×10^5^, maximum injection time 100 ms, isolation window 1.5 *m/z,* and normalized collision energy 35. The resulting data was analyzed in Skyline (version 4.1.0.18169). Precursor and fragment ions selected for PRM assay are summarized in Table S2.

## Purification of recombinant cGAS protein

Recombinant cGAS and mutants were expressed and purified from *Escherichia coli.* The BL21/pLys strain harboring a His6-SUMO tagged and codon-optimized plasmid encoding each of the cGAS proteins was induced with 0.5 mM IPTG at 18°C for 20 hours. Bacteria were sonicated in a lysis buffer of 20 mM HEPES pH 7.5, 500 mM NaCl, 25 mM imidazole, 5 mM β-mercaptoethanol, and 0.2 mM PMSF. After centrifugation at 20,000 g for 60 min, supernatant was incubated with Ni-NTA beads (Qiagen), washed with lysis buffer, and eluted with 20 mM HEPES, pH 7.5, 500 mM NaCl, and 250 mM imidazole. After SUMO protease (Ulp1) digestion at 4°C overnight, untagged cGAS was purified on a HiTrap Heparin column (GE Healthcare) with a gradient of 0.5-1 M NaCl in 20 mM Tris-HCl, pH 7.5. Eluted cGAS protein was concentrated and buffer exchanged to a solution of 20 mM Tris-HCl, pH 7.5, and 150 mM NaCl.

## Immunofluorescence and confocal microscopy

For immunofluorescence imaging, cells seeded in chambered glass slides (Lab-Tek) were first fixed with 4% paraformaldehyde (PFA) in phosphate-buffered saline (PBS) for 15 min. After three PBS washes, cells were permeabilized with 0.2% Triton-X100 in PBS (PBS-T) for 15 min. After three washes in PBS-T, cells were blocked in 5% BSA in PBS-T for 1 hr and stained with the indicated primary antibody in 5% BSA in PBS-T at 4°C overnight. The slides were washed three times with PBS-T before staining with a secondary antibody and 2 μM Hoechst 33342 (Thermo Scientific) in 5% BSA in PBS-T for 1 hr. After three final PBS-T washes, cells were sealed under cover glass in VECTORSHIELD mounting media (Vector Laboratories), and confocal microscopy was performed with an A1R Confocal Microscope (Nikon) with a 60× oil immersed objective. Images were processed in ImageJ.

## Mitochondria isolation and trypsin protection assay

BJ-5ta STING^KO^ cells stably expressing Flag-tagged cGAS or cGAS-ΔN were harvested by trypsin digestion, washed, and swollen in hypertonic buffer on ice for 5 min, and passed through 27g needles for lysis. Lysed cells were centrifuged at 1000 g for 10 min, and the resulting supernatant was further centrifuged at 10,000 g for 10 min. The pellet was resuspended in 200 μL 36% iodixanol solution and overlaid on with 400 μL of 30% iodixanol and 400 μL 10% iodixanol. After centrifugation at 100,000 g for 2 hr, fractions collected from the top to bottom were analyzed by immunoblotting for Tom20 to identify the mitochondria-containing layers. We noticed that some full length cGAS also cosedimented with the mitochondria fraction through this procedure (data not shown), and was used as a control for cGAS-ΔN. 10 μL of the mitochondrial fraction was used for the trypsin protection assay in the presence or absence of 1% NP40. Trypsin was used at 0 μg/μL, 0.025 mg/mL, or 0.05 mg/mL as indicated in fig. S8A. After incubation at 37° C for 40 min, the remaining proteins were boiled in SDS sample buffer and analyzed by immunoblotting.

## Mitochondrial DNA elimination

Elimination of mitochondrial DNA was achieved by overexpression of mitochondrial targeted uracil-N-glycosylase (UNG1-Y147A) in BJ-5ta cells. UNG1 normally excises uracil from DNA. The Y147A mutation at the catalytic active site enables the enzyme to cut thymine as well. Therefore, overexpressing this mutant enzyme in mitochondria result in extensive mtDNA damage that ultimately leads to mtDNA depletion. The expression plasmid (pMA3790, Addgene #70110) was a gift from Mikhail Alexeyev (43). To evaluate the effect of elimination, mitochondrial and nuclear genomic DNA was purified using the QIAamp DNA mini kit (Qiagen). Leu t-RNA (mitochondrial) and DNA polymerase gamma (POLG) were quantified by qPCR to indicate relative abundance of mitochondrial genome and nuclear genome, respectively. qPCR primers: Leu t-RNA (mitochondrial genome): GATGGCAGAGCCCGGTAATCGC and TAAGCATTAGGAATGCCATTGCG; POLG (nuclear genome): AGCGACGGGCAGCGGCGGCGGCA and CCTCCGAGGATAGCACTTGCGGC.

## Split GFP complementation

The G10-Flag-cGAS construct was made by fusing the β-strand 10 of sfGFP MDLPDDHYLSTQTILSKDLN, a linker sequence GTDVGSGGGS, the Flag peptide DYKDDDDK, a short linker TR, and human cGAS. The G10-Flag-cGAS construct was expressed using the pTY lentiviral vector with hygromycin resistance. The cGAS-HA-G11 construct was made by connecting human cGAS, a short linker VD, the HA peptide YPYDVPDYA, a linker sequence GGGSGSGGGSGGGSTS, and the β-strand 11 of sfGFP EKRDHMVLLEYVTAAGITDAS. The cGAS-HA-G11 construct was expressed using the pTY lentiviral vector with blasticidin resistance. The GFP1-9 construct was tagged with the N-terminal myc tag MEQKLISEEDL and a GSGS linker. The GFP1-9 construct was expressed using the pTY lentiviral vector with puromycin resistance. For mitochondrial targeting, these constructs were also tagged in each N-terminus with the Hsp60 signal peptide MLRLPTVFRQMRPVSRVLAPHLTRAY. After simultaneous lentiviral transduction of all three constructs, stable HEK293T cells were selected in 1 μg/mL puromycin, 250 μg/mL hygromycin, and 5 μg/mL blasticidin. Clones with high expression level of all three constructs were selected. GFP formation in the resulting cell lines was observed by confocal fluorescence microscopy.

Recombinant proteins of the three constructs were also expressed and purified from *E. coli.* To test fluorescence complementation in vitro, the three components and Cy3-labelled ISD were mixed at the concentration of 3.5 μM each in a buffer of 20 mM Tris-HCl, pH 7.4, 5 mM MgCl_2_, 0.2 mg/mL BSA, 1 mM ATP, and 1 mM GTP. GFP and Cy3 intensity was measured in clear bottom 96-well plate with a CLARIOstar microplate reader (BMG LABTECH), performing scans at 60 sec intervals for 60 min, with a 2 mm orbital average. At the end of incubation, the level of phase separation was observed under epi-fluorescence microscopy, and cGAMP production was measured as described above. To test the effect of TFAM on cGAS activation, the mixture was supplemented with 2.4 μM recombinant TFAM (Novus Biological) or not.

## in vitro kinase assay

Recombinant cGAS proteins were expressed and purified from *E.coli* as previously described (7). GST-AurB was also expressed and purified from *E.coli.* Briefly, bacterial cultures were induced at OD_600_ = 0.5 with 0.5 mM IPTG and cultured at 25°C for 16 hours. Harvested bacterial pellet was resuspended in a lysis solution containing 50 mM This HCl, pH 8.0, 250 mM NaCl, 1 mM DTT, and protease inhibitor cocktail and sonicated. After centrifugation, cleared lysate was incubated with glutathione sepharose 4B resin at 4°C for 1 hr, washed with lysis solution and eluted in 50 mM This HCl, pH 8.0, 250 mM NaCl, 1 mM DTT, and 20 mM reduced glutathione at 4°C for three times. Combined eluates were dialyzed overnight against 50 mM This HCl, pH 8.0, 250 mM NaCl, 1 mM DTT. To conduct the kinase assay, 0.7 μM recombinant cGAS-Flag was incubated with 0, 0.3, or 0.9 μM GST-AurB in 20 mM Tris pH 7.5, 5 mM MgCl_2_, 5 mM ATP at 30°C for 1 hr. Resulting phosphorylation of cGAS was analyzed by PhosTag electrophoresis.

## RT-qPCR

Total RNA was purified from cells using the Trizol reagent (Thermo Scientific). cDNA was synthesized with the high-capacity cDNA reverse transcription kit (Applied Biosystems). The relative abundance of *ifnβ* or *cxcl10* was quantified by the ΔΔC_T_ method on a QuantStudio 5 Real-Time PCR instrument (Applied Biosystems) using *gapdh* as the internal control. Primers used: *ifnβ*: AGGACAGGATGAACTTTGAC, TGATAGACATTAGCCAGGAG; *cxcl10:* ACGCTGTACCTGCATCAGCA, TTGATGGCCTTCGATTCTGG; *gapdh*: GAGTCAACGGATTTGGTCGT, TTGATTTTGGAGGGATCTCG.

## Supplementary Material

1
