# Supplementary material for: Phosphorylation and Chromatin Tethering Prevent cGAS activation During Mitosis
Source: Science. Author manuscript; Available in PMC 2021 Jun 2. (PMC8171060; doi:10.1126/science.abc5386)
Supplement: 1 [file NIHMS1698933-supplement-1.pdf]

Science AAAS

# Supplementary Materials for

## Phosphorylation and Chromatin Tethering Prevent cGAS activation During Mitosis

Tuo Li, Tuozhi Huang, Mingjian Du, Xiang Chen, Fenghe Du, Junyao Ren, and Zhijian J. Chen*

* Corresponding author. Email: Zhijian.Chen@UTSouthwestern.edu

**This PDF file includes:**

Figs. S1 to S8
Tables S1 to S2

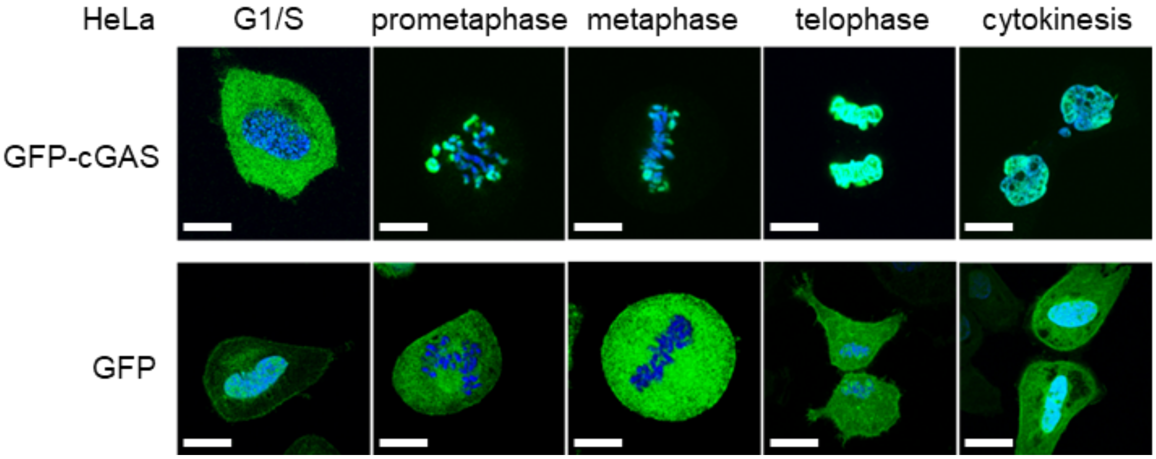


**Fig. S1. cGAS is associated with mitotic chromatin.** cGAS is associated with mitotic chromatin. HeLa cells stably expressing GFP-cGAS or GFP (as a control) were synchronized at G1/S phase with double thymidine blocks and then released for mitosis. Cells at different stages were fixed, stained with DAPI, and visualized by confocal microscopy. Size bars = 10 µm.

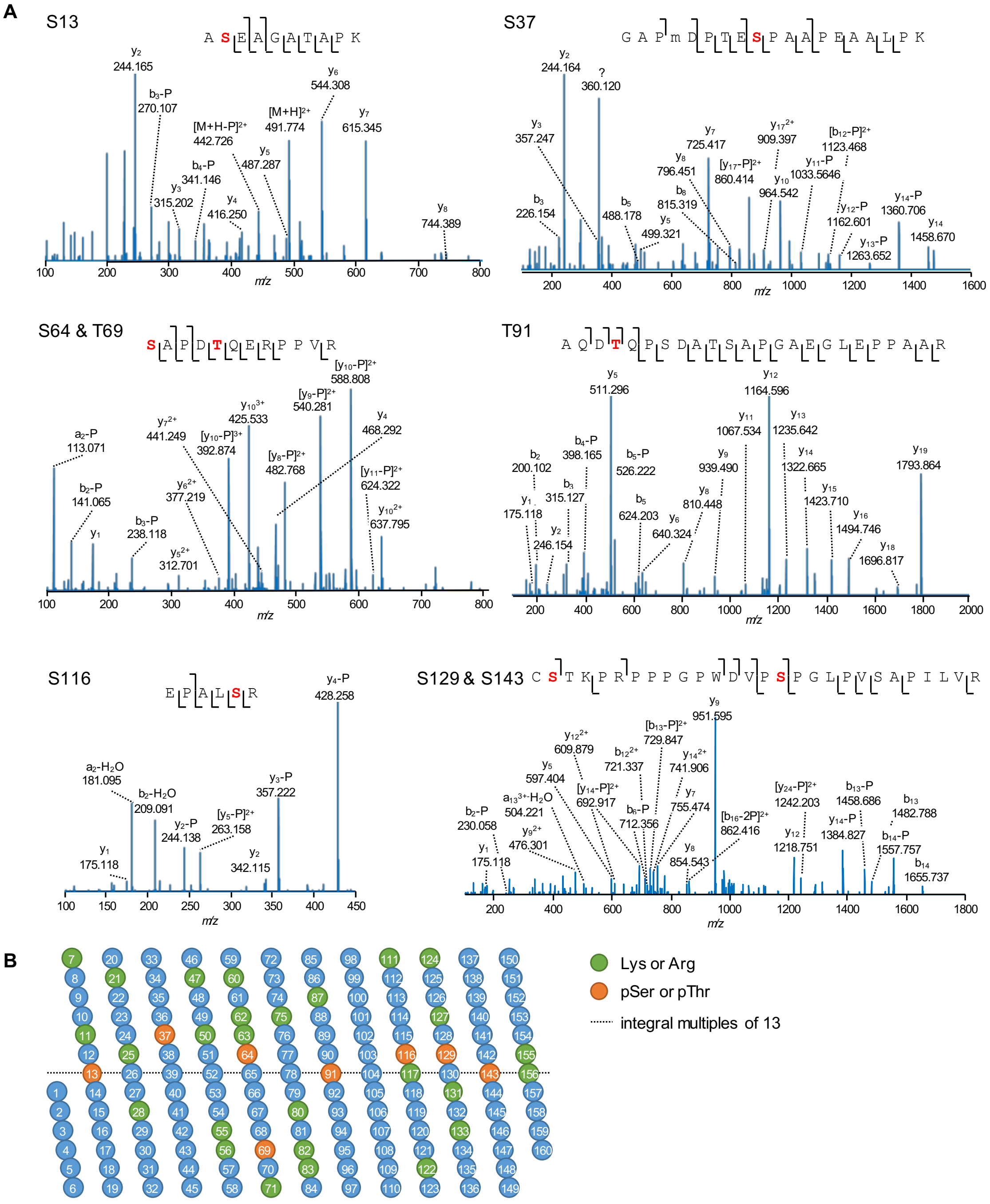


**Fig. S2. Identification of the phosphorylation sites of cGAS isolated from mitotic cells.** Identification of the phosphorylation sites of cGAS isolated from mitotic cells. (**A**) Representative high-energy collisional dissociation (HCD) spectra for phosphorylated peptides of cGAS. (**B**) Distribution of identified phosphorylation sites along the intrinsically disordered N-terminus of cGAS. To highlight the periodicity of the phosphorylation sites (orange dots), the primary sequence is segmented every 13 residues. Green circles denote lysine or arginine residues, which facilitate DNA binding.

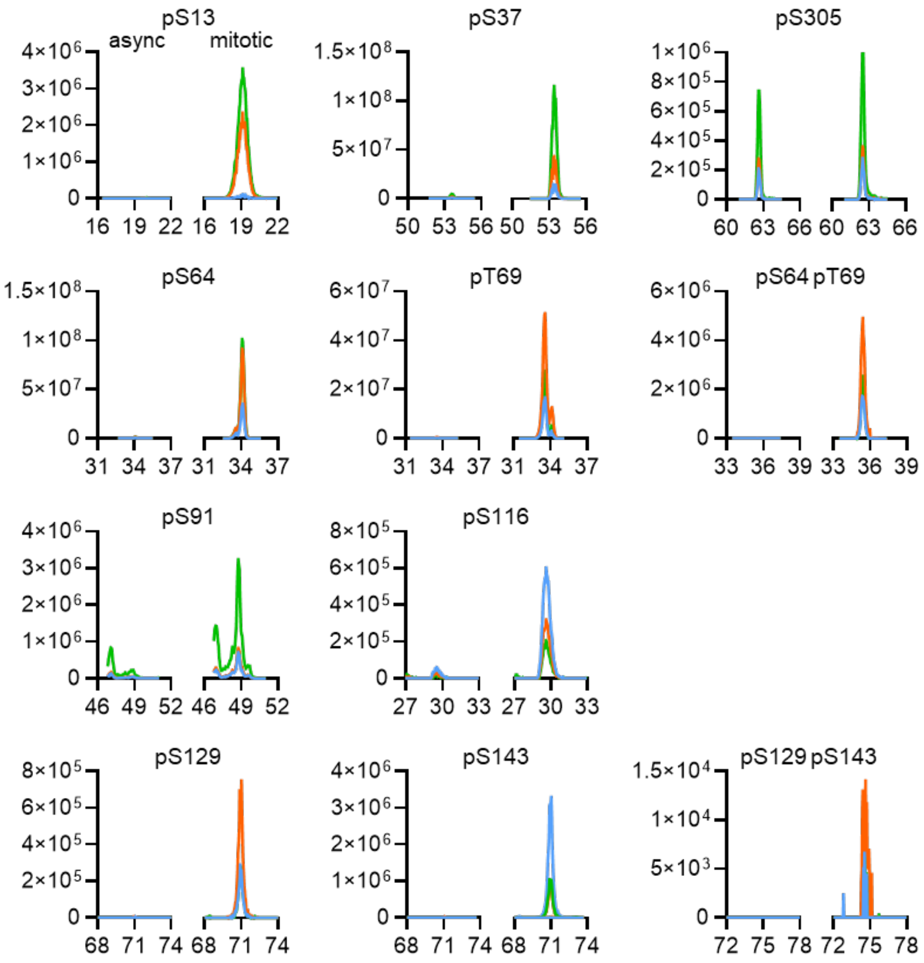


**Fig. S3. Quantification of cGAS phosphorylated peptides in asynchronized and mitotic cells.** Quantification of cGAS phosphorylated peptides in asynchronized and mitotic cells. Representative parallel reaction monitoring (PRM) data show the intensities of top three transition ions (shown in orange, green and blue) selected to quantify cGAS phosphorylated peptides. Data is shown in pairs with matching intensity scales. Each subpanel: (left) asynchronized; (right) mitotic cells.

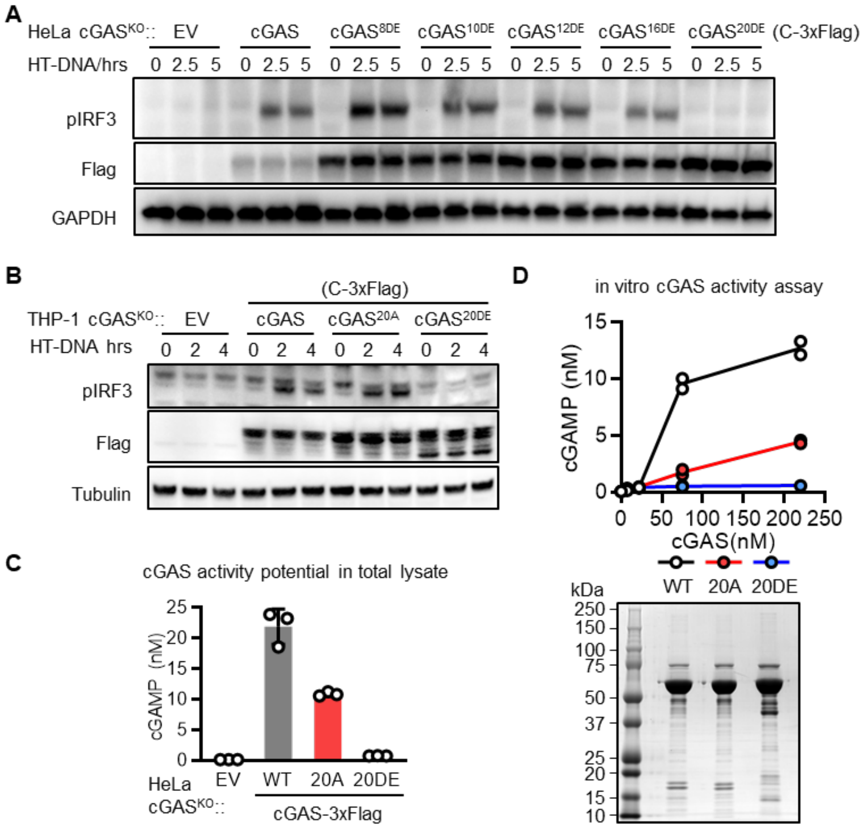


**Fig. S4. Phosphorylation mimicking substitutions at the N-terminus inhibit cGAS activity.** Phosphorylation mimicking substitutions at the N-terminus inhibit cGAS activity. (**A**) HeLa cGAS$^{KO}$ cells stably expressing cGAS or mutants with indicated numbers of serine and threonine residues replaced with aspartate or glutamate, respectively (e.g., cGAS$^{8DE}$ or cGAS$^{20DE}$), were transfected with HT-DNA for the indicated time. Cell lysates were analyzed by immunoblotting with antibodies against indicated proteins. EV: empty vector. (**B**) THP-1 cGAS$^{KO}$ cells stably expressing cGAS, cGAS$^{20A}$, or cGAS$^{20DE}$ were transfected with HT-DNA for the indicated time, followed by immunoblotting. (**C**) HeLa cGAS$^{KO}$ cells stably expressing cGAS, cGAS$^{20A}$, or cGAS$^{20DE}$ were lysed in hypotonic buffer. cGAS activity in the lysate was analyzed in the presence of HT-DNA. Data represent mean ± SD (N=3). (**D**) Wild type cGAS, cGAS$^{20A}$, and cGAS$^{20DE}$ were expressed and purified from *E.coli*. (upper) The purified recombinant proteins were incubated with HT-DNA, ATP, and GTP at 37°C for 1 hour. The level of cGAMP was measured by delivery into PFO-permeabilized THP-1 Lucia cells. (lower) Commassie blue staining of purified recombinant proteins.

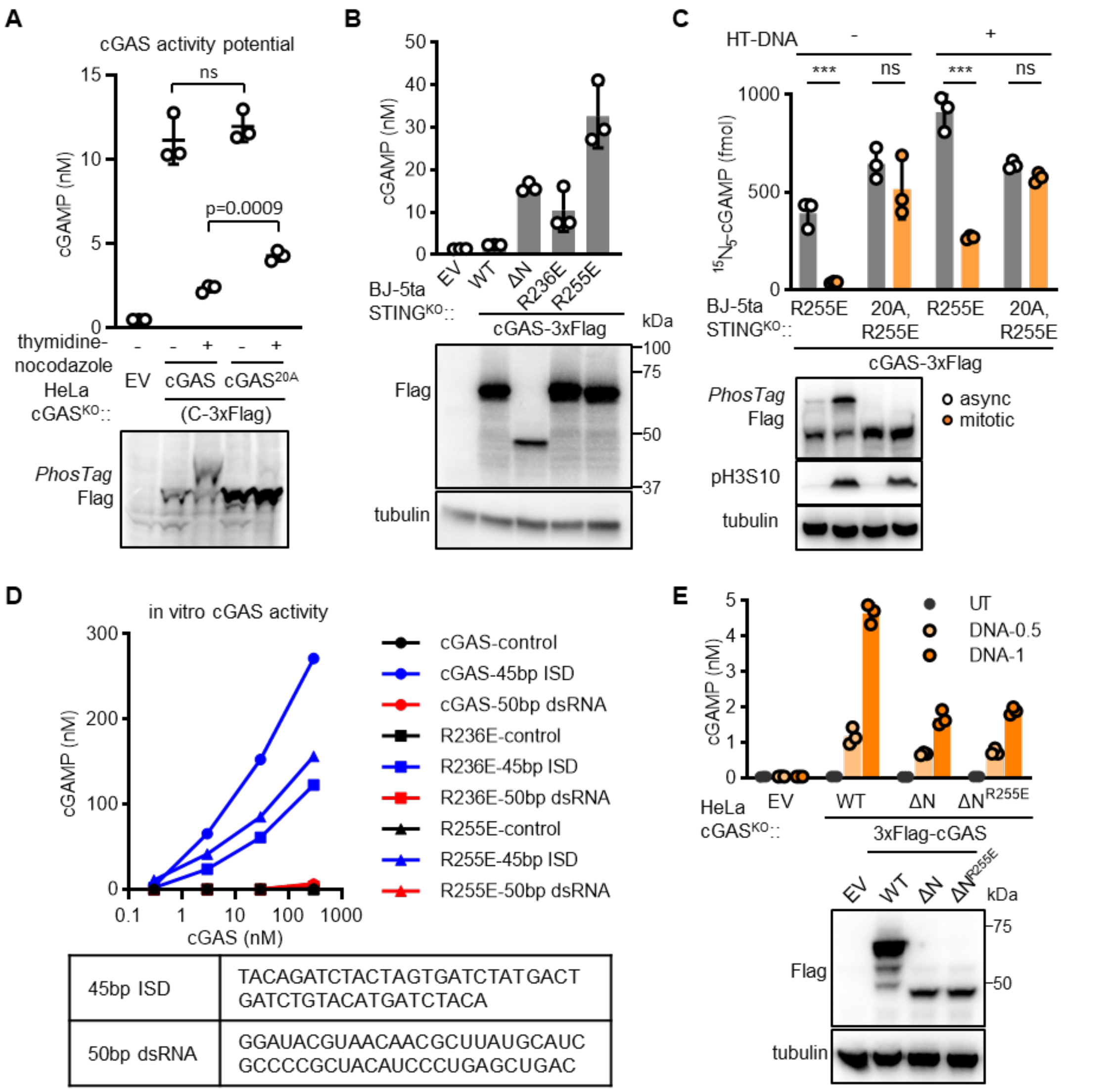


**Fig. S5. The cGAS$^{R236E}$ and cGAS$^{R255E}$ mutants are inhibited by N-terminal phosphorylation during mitosis.** The cGAS$^{R236E}$ and cGAS$^{R255E}$ mutants are inhibited by N-terminal phosphorylation during mitosis. (**A**) cGAS-deficient HeLa cells were reconstituted with C-Flag cGAS, the cGAS$^{20A}$ mutant, or an empty vector (EV) and synchronized with thymidine and nocodazole or left untreated (async). The cells were lysed in hypotonic buffer and cGAS activity in the lysate was measured in the presence of HT-DNA. (lower) Phosphorylation of cGAS was analyzed by PhosTag electrophoresis. (**B**) Human BJ-5ta cells deficient in STING were transduced to stably express C-Flag cGAS and mutants (cGAS-ΔN, cGAS$^{R236E}$, or cGAS$^{R255E}$). EV, empty vector. (top) Endogenous cGAMP levels were measured by delivery to THP-1 Lucia cells. (bottom) Immunoblotting show protein levels of cGAS and mutants. (**C**) BJ-5ta STING$^{KO}$ cells stably expressing C-Flag tagged cGAS$^{R255E}$ or cGAS$^{R255E}$, 20A were synchronized with thymidine and nocodazole or left untreated (async). The cells were lysed in hypotonic buffer and the lysates were incubated with ATP, $^{15}N_5$-GTP, and with or without HT-DNA at 37°C for 1 hour. (top) The level of $^{15}N_5$-cGAMP was measured by quantitative mass spectrometry. Unpaired t-test: ns, not significant; ***, p≤0.001.

(bottom) PhosTag electrophoresis shows the level of cGAS hyperphosphorylation, and immunoblotting shows the level of pH3S10. (**D**) The enzymatic activity of recombinant cGAS, $cGAS^{R236E}$, and $cGAS^{R255E}$ was measured in the absence or presence of 45bp ISD (dsDNA) or 50bp RNA. (lower) Table shows sequences of 45 bp ISD and 50 bp dsRNA used for the assay. (**E**) HeLa $cGAS^{KO}$ cells stably expressing empty vector (EV), N-terminal 3×Flag-tagged cGAS, cGAS-ΔN (ΔN), or $\Delta N^{R255E}$ were transfected with 0.5 or 1 μg/mL HT-DNA for 6 hour or untreated (UT). (top) cGAMP levels after treatment; (bottom) immunoblotting of the indicated proteins. (A, B, C, E) Data represent mean ± SD (N=3). (A, C) Unpaired t-test: ns, not significant; *** $p \leq 0.001$.

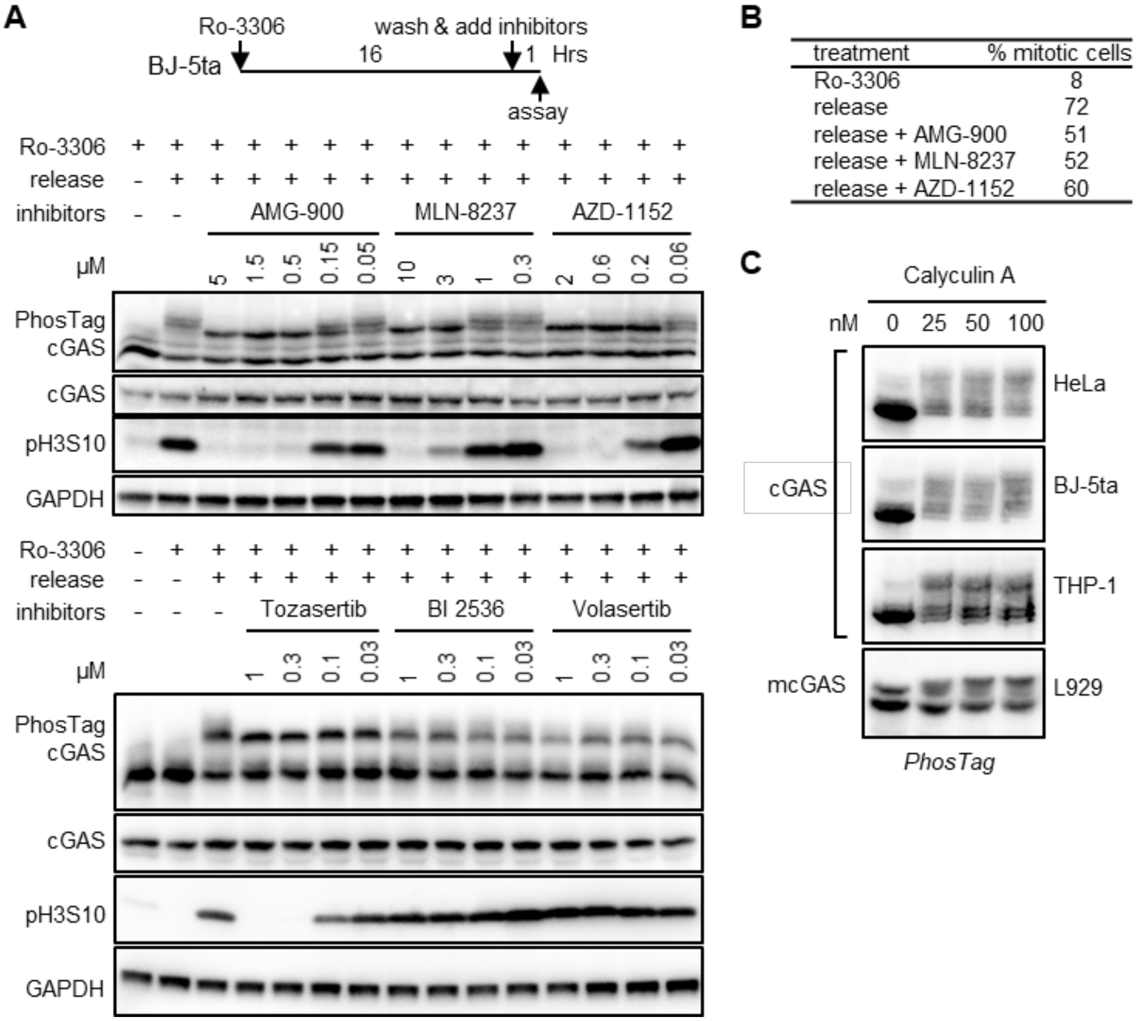


| treatment | % mitotic cells |
|---|---|
| Ro-3306 | 8 |
| release | 72 |
| release + AMG-900 | 51 |
| release + MLN-8237 | 52 |
| release + AZD-1152 | 60 |

**Fig. S6. Aurora kinase B phosphorylates cGAS during mitosis.** Aurora kinase B phosphorylates cGAS during mitosis. (**A**) BJ-5ta cells were synchronized with 10 µM Ro-3306 for 16 hours and released into mitosis for 1 hour, in the absence or presence of aurora kinase inhibitors (AMG-900, MLN-8237, AZD-1152, or Tozasertib) or polo-like kinase inhibitors (BI 2536 or Volasertib) at the indicated concentrations. Phosphorylation of cGAS was analyzed by PhosTag electrophoresis. Phosphorylation of histone 3 serine 10 (pH3S10) was analyzed by immunoblotting. (**B**) Percentages of mitotic cells at the time of assay for (A) are shown for the highest indicated concentrations of aurora kinase inhibitors, showing that these inhibitors did not block mitosis. (**C**) PhosTag electrophoresis show the phosphorylation status of endogenous cGAS in different cell lines treated with calyculin A at indicated concentrations for 30 min. mcGAS: mouse cGAS.

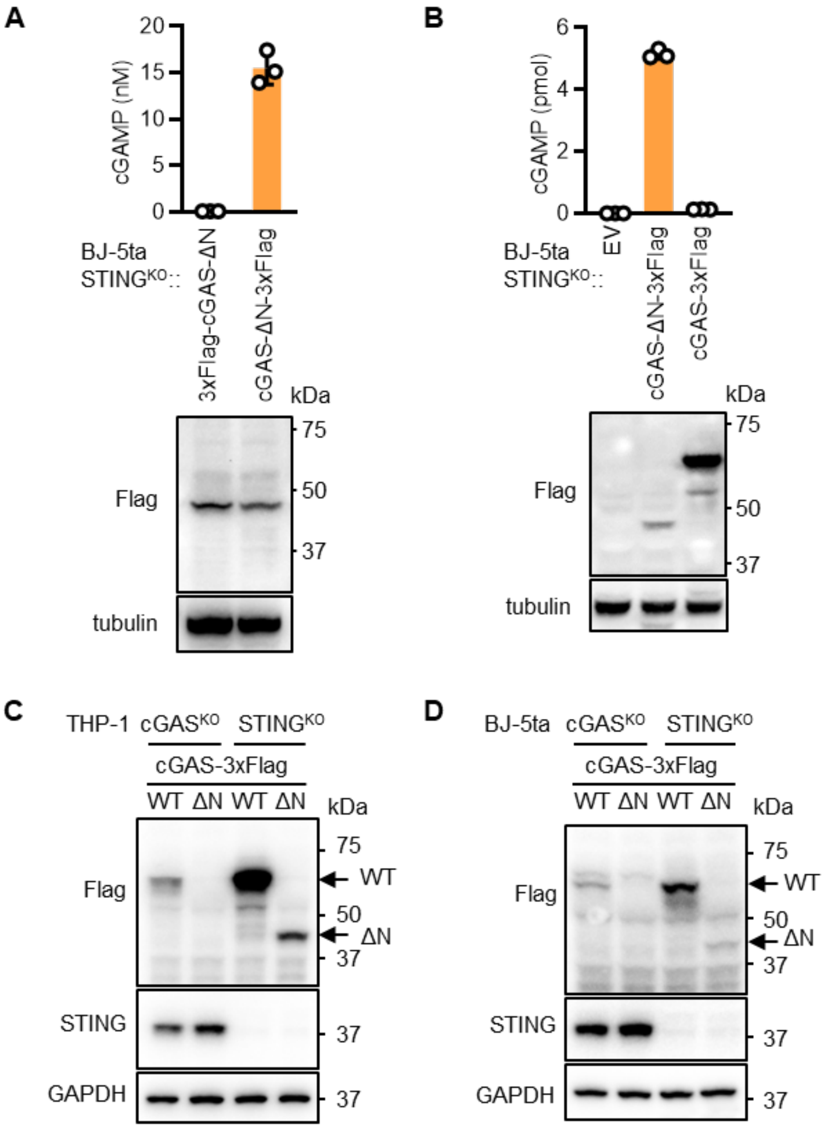


**Fig. S7. cGAS-ΔN is de-repressed and not tolerated in STING-expressing cells.** cGAS-ΔN is de-repressed and not tolerated in STING-expressing cells. (**A**) (top) Endogenous cGAMP levels in BJ-5ta STING$^{KO}$ cells stably expressing cGAS-ΔN that was tagged with N- or C-terminal 3×Flag. (bottom) Immunoblotting showing the expression levels of cGAS-ΔN proteins. (**B**) Endogenous cGAMP levels in BJ-5ta STING$^{KO}$ cells stably expressing C-Flag tagged full-length cGAS or cGAS-ΔN, or empty vector (EV) control. (A-B) Data represent mean ± SD (N=3). (**C**) THP-1 cGAS$^{KO}$ or THP-1 STING$^{KO}$ cells were transduced by lentiviruses to express C-terminal 3×Flag tagged cGAS (WT) or cGAS-ΔN (ΔN). After antibiotic selection, the level of ectopically expressed cGAS was analyzed by immunoblotting. (D) Similar to (C), except that BJ-5ta cGAS$^{KO}$ and BJ-5ta STING$^{KO}$ cells were used.

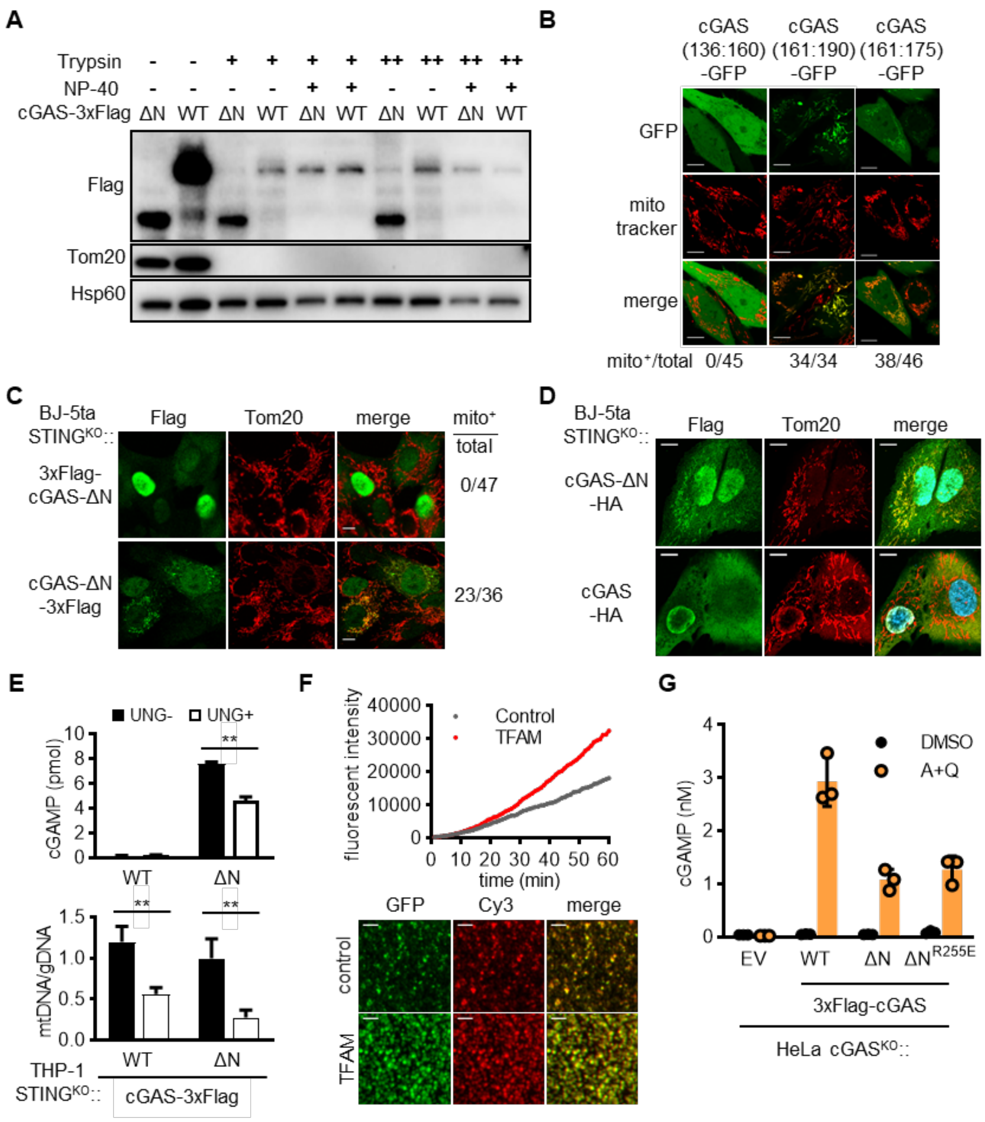


**Fig. S8. cGAS-ΔN is activated by mitochondrial DNA.** cGAS-ΔN is activated by mitochondrial DNA. (**A**) THP-1 STING$^{KO}$ cells stably expressing C-terminal 3×Flag tagged cGAS (WT) or cGAS-ΔN (ΔN) were lysed in a hypotonic buffer. Crude mitochondria were isolated by iodixanol density gradient ultracentrifugation and digested with trypsin (+: 0.025 mg/ml; ++: 0.05 mg/ml) in the presence or absence of 1% NP-40 and analyzed by immunoblotting with antibodies specific for Flag, Tom20 (a mitochondria outer membrane protein), or Hsp60 (a mitochondria matrix protein). (**B**) Confocal microscopy images show the subcellular distribution of GFP that is N-terminally tagged with the selected cGAS peptides 136-160, 161-190, or 161-175. (**C**) Immunofluorescence images show the subcellular localization of N- or C-terminal 3×Flag tagged cGAS-ΔN in comparison to that of Tom20. (**D**) Immunofluorescence images show the localization of C-terminal HA-tagged cGAS or cGAS-ΔN stably expressed in BJ-5ta STING$^{KO}$ cells. (B-D) Numbers on the bottom (B) or to the right (C and D) of the micrographs show fraction of cells with mitochondrial localization of cGAS. Size bars = 10 μm. (**E**) THP-1 STING$^{KO}$ cells stably expressing C-terminal

3×Flag cGAS or cGAS-ΔN (-UNG) were transduced to express the mitochondria-localized uracil-N-glycosylase Y147A mutant (+UNG). (top) Levels of endogenous cGAMP in the cells. (bottom) Relative abundance of mtDNA measured by qPCR normalized to genomic DNA. Unpaired t-test: **, p≤0.01. (**F**) The in vitro split GFP complementation assay for cGAS was conducted in the absence or presence of recombinant TFAM protein. (upper) Total GFP fluorescent intensity measured over time; (bottom) fluorescent microscopy images show assembly of cGAS-DNA droplets at the end of assay. (**G**) HeLa $cGAS^{KO}$ cells stably expressing empty vector (EV), N-terminal 3×Flag-tagged cGAS, cGAS-ΔN (ΔN), or $ΔN^{R255E}$ were treated with 10 μM ABT-737 + 20 μM Q-VD-OPH (A+Q) or with DMSO. cGAMP levels were measured after 6-hour treatment. (E and G) Data represent mean ± SD (N=3). Unpaired t-test: **, p≤0.01.

| Site | Peptide Sequence | Start Stop | z | mass | | Δmass | | Scores | | | Peptide Identification Probability | Localization Probability |
|---|---|---|---|---|---|---|---|---|---|---|---|---|
| | | | | Actual | Observed | ΔAMU | PPM | Xcorr | ΔCn | X!Tandem | | |
| Ser13 | AsEAGATAPK | 12-21 | 2 | 981.4159 | 491.7152 | -0.001 | -1.1 | 2.06 | 0.61 | 3.89 | 100% | 100% |
| Ser37 | GAPmDPTEsPAAPEAALPK | 29-47 | 2 | 1944.8479 | 973.4312 | -0.006 | -3.3 | 2.77 | 0.7 | 4.51 | 100% | 100%, 100% |
| Ser64 & Thr69 | sAPDtQERPPVR | 64-75 | 3 | 1511.6141 | 504.8786 | -0.003 | -2.1 | 2.1 | 0.55 | 4.38 | 100% | 100%, 100% |
| Thr91 | AQDtQPSDATSAPGAEGLEPPAAR | 88-111 | 2 | 2416.0504 | 1209.0325 | -0.004 | -1.8 | 2.97 | 0.78 | 16.33 | 100% | 100% |
| Se116 | EPALsR | 112-117 | 2 | 752.3338 | 376.6684 | -0.002 | -5.8 | 1.59 | na | na | na | 100% |
| Ser143 | cSTKPRPPPGPWDVPsPGLPVSAPILVR | 128-155 | 3 | 3056.5664 | 1019.8627 | -0.001 | -0.4 | 3.17 | 0.75 | 10.18 | 100% | 100% |
| Ser129 & Ser143 | csTKPRPPPGPWDVPsPGLPVSAPILVR | 128-155 | 3 | 3136.5353 | 1046.5190 | 0.001 | 0.4 | 3.73 | 0.8 | 11.92 | 100% | 100%, 100% |
| S305 | GGsPAVTLLISEK | 303-315 | 2 | 1350.6756 | 676.3451 | -0.004 | -3.1 | 2.08 | 0.63 | 4.72 | 100% | 100% |

**Table S1. Mass spectra for identification of cGAS phosphorylation sites.** Mass spectra for identification of cGAS phosphorylation sites. z: charge; ΔAMU: mass error in atomic mass unit; PPM: relative mass error in parts per million; Xcorr: SEQUEST correlation score for evaluating peptide-spectrum matches (PSM); ΔCn: difference in scores of the best fit and the second best fit; X!Tandem: PSM scores by X!Tandem.

| Peptide Sequence | Precursor | z | PRM Products, mass and charges |
|---|---|---|---|
| ASEAGATAPK | 451.7325 | 2 | y6+, y7+, y8+<br>544.3089, 615.3461, 744.3886 |
| -p-------- | 491.7157 | 2 | y6+, y7+, y8+<br>544.3089, 615.3461, 744.3886 |
| GAPmDPTESPAAPEAALPK | 925.4537 | 2 | y7+, y10+, y11+<br>725.4192, 964.5462, 1051.5782 |
| --------p---------- | 965.4368 | 2 | y7+, y10+, y11-98+<br>725.4192, 964.5462, 1033.5677 |
| SAPDTQERPPVR | 451.5688 | 3 | Y8++, y9++, y10++<br>491.7751, 549.2885, 597.8149 |
| P----------- | 478.2242 | 3 | Y8++, y9++, y10++<br>491.7751, 549.2885, 597.8149 |
| --------p--- | 478.2242 | 3 | y8-98++, y9-98++, y10-98++<br>482.7698, 540.2833, 588.8096 |
| P-------p--- | 504.8796 | 3 | y8-98++, y9-98++, y10-98++<br>482.7698, 540.2833, 588.8096 |
| AQDTQPSDATSAPGAEGLEPPAAR | 779.7033 | 3 | y5+, y8+, y12+, b4+, b5+<br>511.2987, 810.4468, 1164.6008, 416.1776, 544.2362 |
| ---p-------------------- | 806.3587 | 3 | y5+, y8+, y12+, b4-98+, b5-98+<br>511.2987, 810.4468, 1164.6008, 398.1670, 526.2256 |
| EPALSR | 336.6874 | 2 | y2+, y3+, y4+<br>262.1510, 375.2350, 446.2722 |
| ----p- | 376.6706 | 2 | y2-98+, y3-98+, y4-98+<br>244.1404, 357.2245, 428.2616 |
| CSTKPRPPPGPWDVPSPGLPVSAPILVR | 745.1575 | 4 | b2+, b3+, b6+, b13+, b14+<br>248.0700, 349.1176, 730.3665, 738.8563, 788.3905 |
| -p-------------------------- | 765.1491 | 4 | b2-98+, b3-98+, b6-98+, b13-98+, b14-98+<br>230.0594, 331.1071, 712.3559, 729.8510, 779.3852 |
| ---------------p------------ | 765.1491 | 4 | b2+, b3+, b6+, b13+, b14+<br>248.0700, 349.1176, 730.3665, 738.8563, 788.3905 |
| -p-------------p------------ | 785.1406 | 4 | b2-98+, b3-98+, b6-98+, b13-98+, b14-98+<br>230.0594, 331.1071, 712.3559, 729.8510, 779.3852 |
| GGSPAVTLLISEK | 636.3639 | 2 | y3+, y7+, y8+<br>363.1874, 803.4873, 902.5557 |
| --p---------- | 676.3471 | 2 | y3+, y7+, y8+<br>363.1874, 803.4873, 902.5557 |

**Table S2. cGAS peptides, precursor ions and fragment Ions selected for parallel reaction monitoring (PRM).** cGAS peptides, precursor ions and fragment Ions selected for parallel reaction monitoring (PRM).
